# *HLA-DRB1* polymorphisms and alopecia areata disease risk: A systematic review and meta-analysis

**DOI:** 10.1097/MD.0000000000011790

**Published:** 2018-08-10

**Authors:** Conghua Ji, Shan Liu, Kan Zhu, Hongbin Luo, Qiushuang Li, Ying Zhang, Sijia Huang, Qing Chen, Yi Cao

**Affiliations:** a Center of Clinical Evaluation, Zhejiang Hospital of Traditional Chinese Medicine, Hangzhou, Zhejiang, China; b Institute for Regenerative Cures, Department of Dermatology, University of California Davis School of Medicine, Sacramento, CA, USA; c Department of Dermatology, Zhejiang Hospital of Traditional Chinese Medicine, Hangzhou, Zhejiang; d Department of Epidemiology, School of Public Health, Southern Medical University, Guangzhou, Guangdong, China.

**Keywords:** alopecia areata, *DRB1*, human leukocyte antigen, odds ratio, polymorphism, risk

## Abstract

**Background:**

Published studies have reported conflicting and heterogeneous results regarding the association between human leukocyte antigen (*HLA*)-*DRB1* polymorphisms and alopecia areata (AA). This study aimed to review and quantitatively analyze the association between *HLA*-*DRB1* polymorphisms and AA.

**Methods:**

In this study, all relevant publications were searched through December 2016. Odds ratios (ORs) and confidence intervals (CIs) for comparisons between case and control groups were calculated. Stata 14.0 software was used to perform statistical analysis. This research does not require formal ethical approval because the data used in this analysis do not involve personal information and thus do not affect privacy.

**Results:**

Twelve articles were identified. For *HLA-DRB1∗04* and *HLA-DRB1∗16* polymorphisms, the OR (95% CIs) was 1.49 (1.24–1.78) and 1.61 (1.08–2.41), and *P* was <.01 and <.01, respectively. For *HLA-DRB1∗0301, HLA-DRB1∗09*, and *HLA-DRB1∗13* polymorphisms, the OR (95% CIs) was 0.42 (0.28–0.63), 0.74 (0.55–0.99), and 0.62 (0.40–0.98), and *P* was <.01, <.01, and <.01, respectively. Statistical evidence revealed no publication bias (*P* > .05).

**Conclusion:**

The present meta-analysis suggested that *HLA-DRB1∗04* and *HLA-DRB1∗16* polymorphisms might be associated with increased AA risk, while *HLA-DRB1∗0301*, *HLA-DRB1∗09*, and *HLA-DRB1∗13* polymorphisms might decrease the AA risk. Studies with adequate methodological quality on gene–gene and gene–environment interactions are needed to validate the results in the future.

## Abstract 2

Supplemental Digital Content is available in the text

## 1 Introduction

Alopecia areata (AA) is a cell-mediated autoimmune disease causing an unpredictable hair loss with no overt epidermal changes. The lifetime risk of AA is estimated to be 1.7%. It affects both sexes and people of all races, but is more prevalent in children.^[1]^ In AA, abnormal immune damage leads to round or oval patches, which may advance to all scalp hair (alopecia totalis) or all body hair (alopecia universalis).^[2]^

Human leukocyte antigen (HLA)-*DRB1* polymorphisms have been discussed in many types of autoimmune diseases, for instance, aplastic anemia,^[3]^ systemic lupus erythematosus and lupus nephritis,^[4]^ Vogt–Koyanagi–Harada disease,^[5]^ and multiple sclerosis.^[6]^ As one of the autoimmune diseases which caused by several major susceptibility genes, AA is genetically associated with alleles of HLA in different ethnic groups.^[7]^ CD4+ lymphocytes play an important role in AA inflammatory processes. They have been proposed to recognize the antigen and major histocompatibility complex (MHC) class II complexes on macrophages and Langerhans cells, and the expression may be induced on other nucleated cells, leading to AA.^[8,9]^ It is noticed that *HLA-DR* and *HLA-DQ* alleles are responsible for presenting the antigen to CD4+ T cells.^[10]^

One genome-wide association study (GWAS) discussed the relationship between HLA and AA.^[1]^ It revealed HLA-DR as a key etiologic driver. The study indicated *HLA-DRB1∗04:01* polymorphisms as the potential risk factor for AA (OR = 1.64). GWAS explored the genetic architecture of complex diseases, but was limited in detecting any other kinds of genetic variants such as deletions associated with a high percentage of autoimmune diseases.^[11]^

Previous individual studies have been concerned with the association between *HLA-DRB1* polymorphisms and AA. Three studies indicated *HLA-DRB1∗04* allele as a risk factor for the development of AA.^[12–14]^ However, the results were inconsistent with the findings of other studies.^[15–20]^ Moreover, one study suggested a lower occurrence of *HLA-DRB1∗15* polymorphisms in AA,^[21]^ whereas others found no association.^[13,15,16,18–20]^

A number of conflicting studies have reported the relationship between *HLA-DRB1* polymorphisms and AA risk in small samples,^[12–23]^ but no definite consensus existed. Therefore, this meta-analysis aimed to examine the relationship between *HLA-DRB1* polymorphisms and AA. Since a single study might have been underpowered to clarify the genes with AA risk, the purpose of this study was to increase the statistical power and evaluate the evidence from studies by summarizing it quantitatively with a meta-analytic approach.

## 2 Materials and methods

This study was performed following the standards of the Preferred Reporting Items for Systematic Reviews and Meta-analyses (PRISMA) criteria^[24]^ (Supplemental Table 1) and the recommendations of the Cochrane Collaboration.^[25]^ A protocol for this systematic review has been published in PROSPERO with the registration number CRD42015023718 (Supplemental File 1).

### 2.1 Search strategy

We carried out an electronic search of multiple databases, including PubMed, Embase, Cochrane database, Chinese China National Knowledge Infrastructure, Chinese Biomedical Literature Database, Wang Fang, and Chinese Social Sciences Citation Index, through December 2016 for all studies on the association between HLA polymorphisms and AA by using the following keywords (“Alopecia areata” or “nonscarring hair loss” or “ophiasis” or “alopecia celsi” or “alopecia universalis” or “alopecia totalis”) and (“human leukocyte antigen” or “HLA” or “major histocompatibility complex” or “DRB1” or “MHC”) (Supplemental Table 2). No language restrictions were imposed in this research. We also searched the references of the included studies and e-mailed the study authors to identify additional studies and collect missing data.

### 2.2 Inclusion and exclusion criteria

The inclusion criteria were as follows: studies concerned with the association between *HLA-DRB1* polymorphisms and AA; and sufficient data on odds ratio (OR) with a 95% confidence interval (CI).

The exclusion criteria were as follows: reviews, comments, editorials, or basic science or animal studies; genotype frequency not revealed or the relevant data not obtained by contacting authors; and duplicate studies.

### 2.3 Study selection

Two review authors initially screened the titles and abstracts independently. The full text versions of any studies of potential relevance were retrieved and examined carefully according to inclusion and exclusion criteria. Only the most recent study was included when there were overlapping data or even repeating data. Any discrepancies were adjudicated by regular conferences involving the third reviewer (Prof Chen). She downloaded the full text of the inconsistent studies and discussed step by step according to the inclusion and exclusion criteria.

### 2.4 Data extraction

Data extraction was performed independently by 2 investigators using a predetermined extraction form. The third participant was consulted for discussion to reach an agreement concerning discrepancies. The following items were extracted from each study: first author's last name, publication year, country, the Newcastle-Ottawa Scale (NOS), numbers of cases and controls, gene detection method, genes involved, and frequency of *HLA-DRB1* alleles.

### 2.5 Quality assessment for individual studies

A scoring system based on the NOS was used to determine the quality of each study. Items assessed included selection, comparability of cases/controls, and exposure. The score of overall quality ranged from 0 to 9. The NOS score was divided into 3 levels (high quality, score ≥7; moderate quality, 4 ≤ score < 7; low quality, score >4). Disagreements were settled as described earlier.

### 2.6 Statistical analysis

All statistical analyses were conducted using Stata 14.0 (Stata Corporation, TX). Dichotomous data were reported as OR (calculated by the *χ*^2^ test). The pooled ORs and the 95% CIs used for assessing the strength of association were determined by the *Z* test. Heterogeneity across studies was checked by the Cochran *Q* statistic and the *I*^2^ test.^[26]^ If a 2-sided *P* value <.05 was considered as statistically significant, then a random-effects model was used (shown as “D + L”).^[27]^ Otherwise, a fixed-effects model was applied (shown as “M-H”).^[28]^ When *I*^2^ was >50% indicating high heterogeneity, subgroup analyses were used. Subgroup analyses were performed by area to reveal whether it could lead to heterogeneity. Meta-regression was used to reveal whether continent, country, or NOS score could lead to heterogeneity.

A sensitivity analysis was performed by sequential omission of individual studies to evaluate the stability of outcomes.^[29]^ Harbord^[30]^ and Egger^[31]^ tests were conducted to evaluate the publication bias with a *P* value <.05 for considering statistical significance. If publication bias was indicated with statistical significance, a trim-and-fill analysis was performed.^[32]^

## 3 Results

### 3.1 Study characteristics

**Figure 1:**
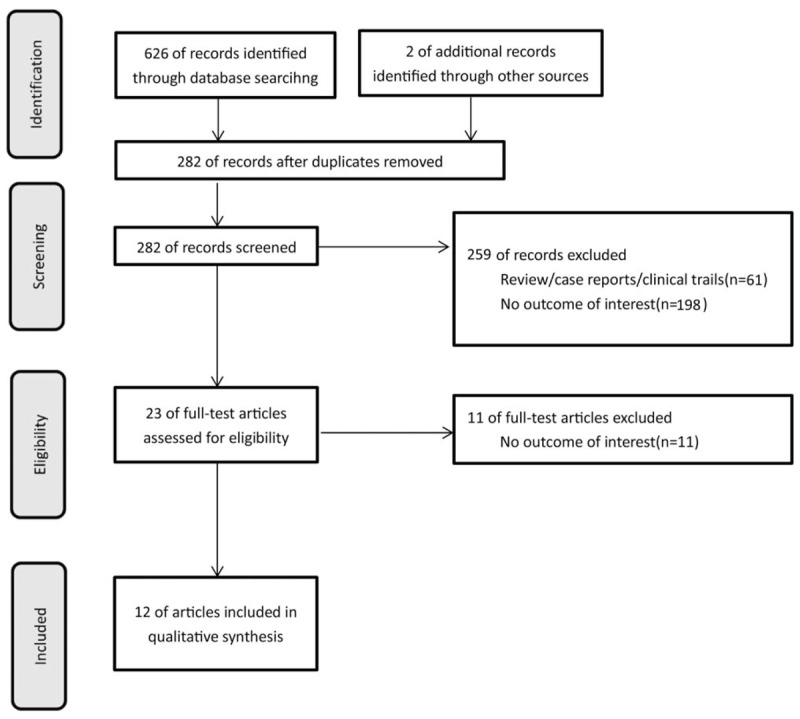
Flow diagram of the study selection process.

We conducted this study under PRISMA statement (Fig. 1). Through literature searches, 626 studies discussed the association of HLA polymorphism and AA. After reading titles and abstracts, 22 studies were identified. Unfortunately, 10 articles were eliminated due to some reasons. The Supplemental Table 3 lists the reasons for the exclusion of these studies. Finally, 12 studies^[12–23]^ consisting 1283 cases and 32,343 controls were included, 2 of which were graduation theses of postgraduate students.^[13,14]^ Zhang et al^[19]^ included 121 cases and 24,930 controls, which accounted for huge different sample sizes in 2 groups. Table 1 lists the included studies and their main characteristics. These studies covered Europe, Asia, America, and Africa. The average NOS score was 5.08, which revealed that the methodological quality was of average level (Table 1 and Supplemental Table 4). Of the 12 studies, 2 were of high quality^[14,16]^ and 10 of moderate quality^[12,13,15,17–23]^ (Supplemental Table 4).

**Table 1:**
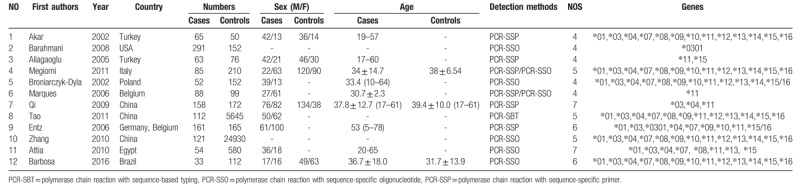
Characteristics of studies included in the meta-analysis.

### 3.2 Quantitative synthesis

Table 2 lists the main results of the meta-analysis. In total, 13 *HLA-DRB1* allele families and 1 specific allele were extracted from the studies to investigate their relationships to AA.

**Table 2:**
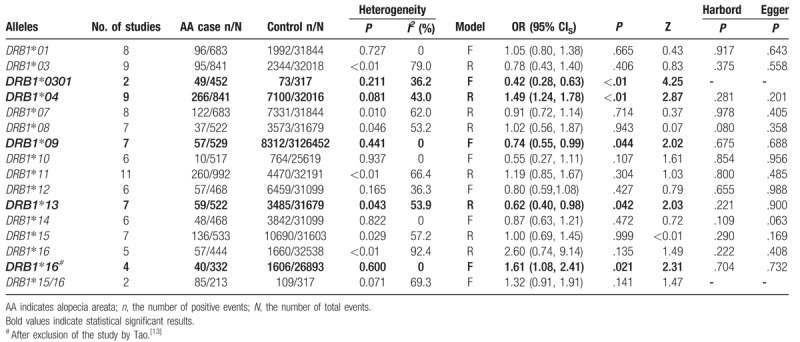
Meta-analysis of associations between *HLA-DRB1* alleles and alopecia areata.

**Figure 2:**
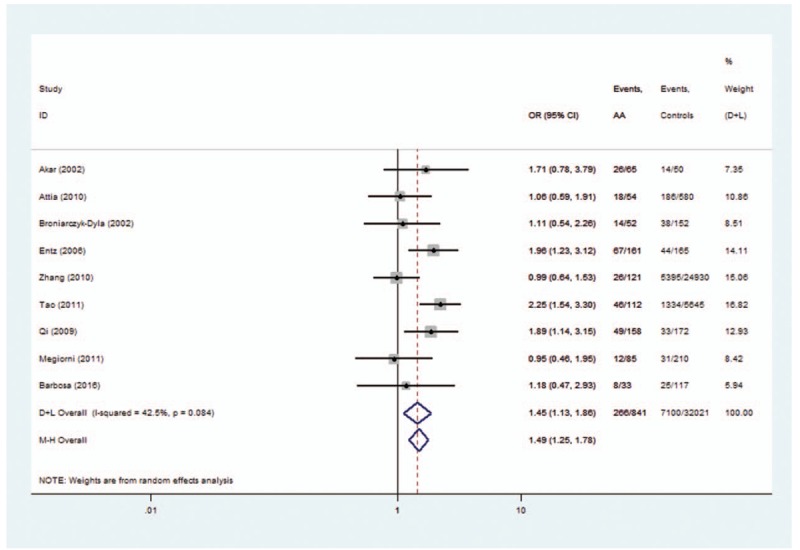
Forest plot of HLA-DRB1∗04 polymorphism and alopecia areata.

**Figure 3:**
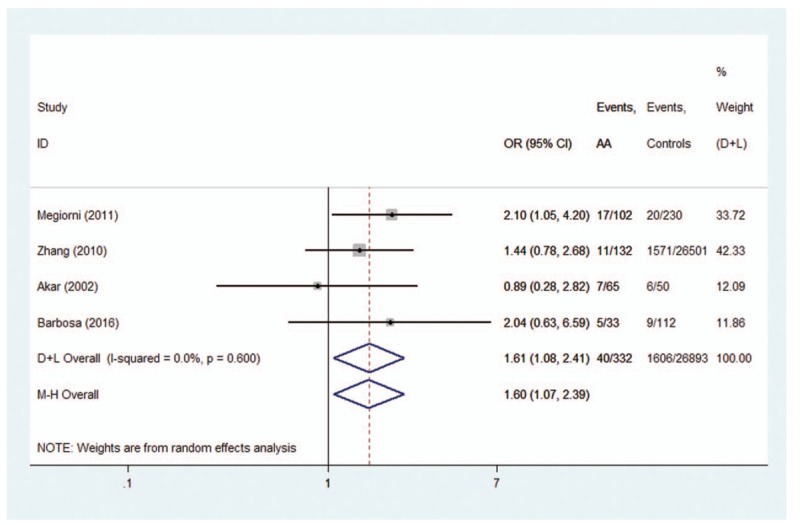
Forest plot of HLA-DRB1∗16 polymorphism and alopecia areata.

Two allele families (*HLA-DRB1∗04* and *HLA-DRB1∗16*) conferred a significantly increased risk. For *HLA-DRB1∗04* polymorphisms, the analysis of the pooled data of 8 case-control studies^[9–16,20]^ revealed a significant increase in frequency (31.6% compared with 22.2% in controls), with an evidence of heterogeneity (*I*^2^ = 43.0%, *P* = .081). A random-effects model was used for calculating OR. Overall OR (95% CIs) was 1.49 (1.24–1.78) with *P* < 0.01 (Fig. 2). For *HLA-DRB1∗16* polymorphisms, the analysis of the pooled data of 4 case-control studies revealed a significant increase in frequency (12.0% compared with 6.0% in controls), with no evidence of heterogeneity (*I*^2^ = 0.0%, *P* = .600). A fixed-effects model was used for calculating OR. Overall OR (95% CIs) was 1.60 (1.07–2.39) with *P* < .05 (Fig. 3).

**Figure 4:**
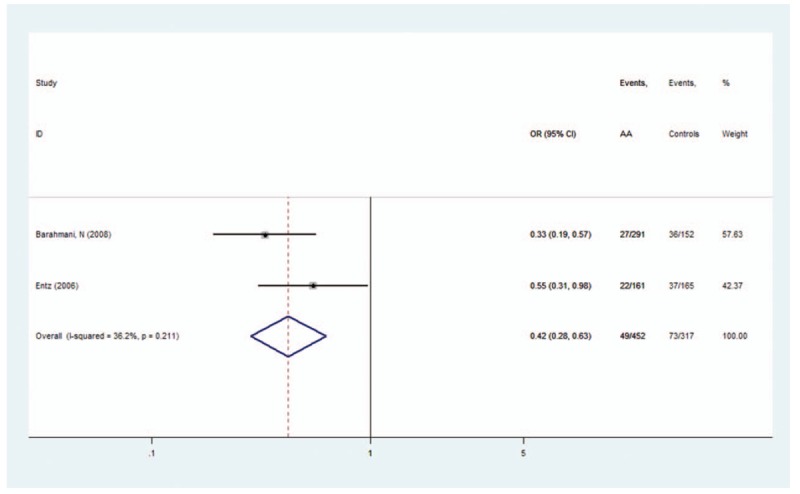
Forest plot of HLA-DRB1∗0301 polymorphism and alopecia areata.

**Figure 5:**
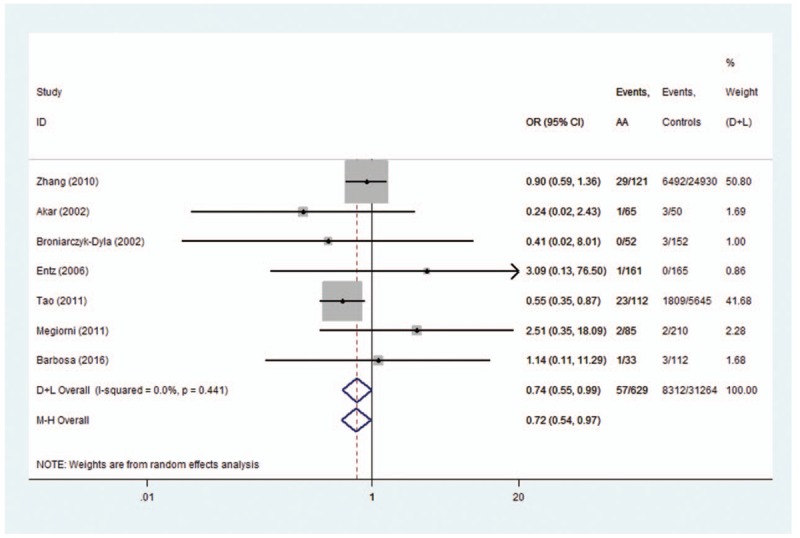
Forest plot of HLA-DRB1∗09 polymorphism and alopecia areata.

**Figure 6:**
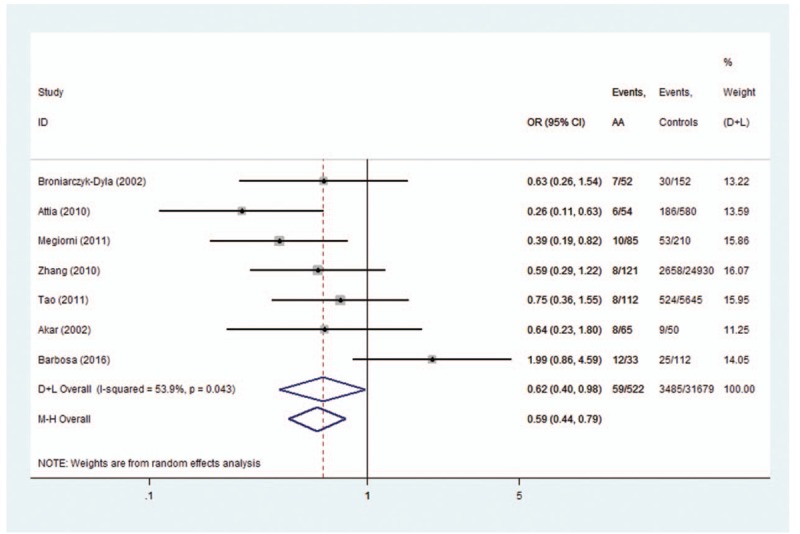
Forest plot of HLA-DRB1∗13 polymorphism and alopecia areata.

*HLA-DRB1∗0301*, *HLA-DRB1∗09*, and *HLA-DRB1∗13* polymorphisms conferred a significant protective effect for AA. A low heterogeneity for *HLA-DRB1∗0301* (*I*^2^ = 36.2%, *P* = .211), *HLA-DRB1∗09* (*I*^2^ = 0%, *P* = .441), and *HLA-DRB1∗13* (*I*^2^ = 53.9%, *P* = .043) polymorphisms was observed. A fixed-effects model was used for calculating OR for *HLA-DRB1∗0301*,*∗09* and a random-effects model was used for calculating OR for *HLA-DRB1∗13*. The OR (95% CIs) was 0.42 (0.28–0.63) for *HLA-DRB1∗0301* (Fig. 4), 0.74 (0.55–0.99) for *HLA-DRB1∗09* (Fig. 5), and 0.62 (0.40–0.98) for *HLA-DRB1∗13* polymorphisms (Fig. 6).

For *HLA-DRB1∗01*, *DRB1∗03*, *DRB1∗07*, *DRB1∗08*, *DRB1∗10*, *DRB1∗11*, *DRB1∗12*, *DRB1∗14*, *DRB1∗15*, and *DRB1∗15/16* alleles, no evidence of association in statistics between *HLA-DRB1* polymorphisms and AA was found (Table 2 and Supplemental File 2).

### 3.3 Subgroup analysis

**Figure 7:**
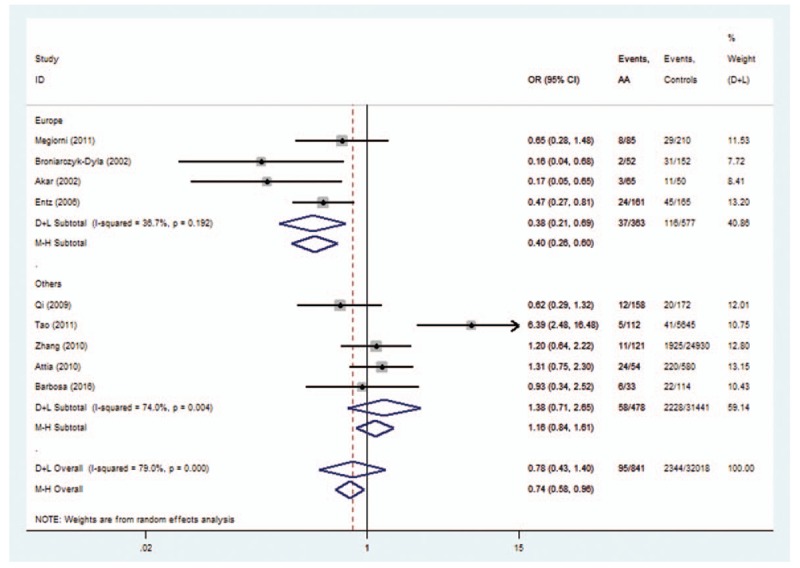
Forest plot of HLA-DRB1∗03 polymorphism and alopecia areata.

**Figure 8:**
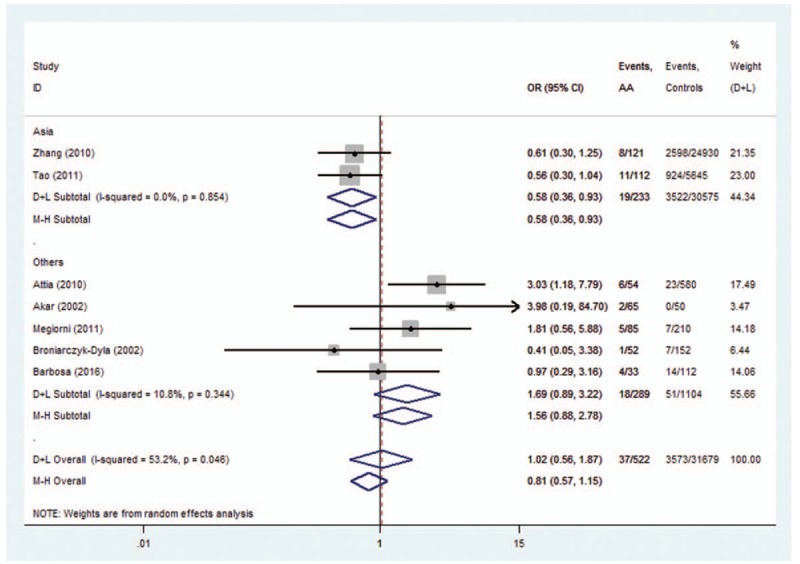
Forest plot of HLA-DRB1∗08 polymorphism and alopecia areata.

**Figure 9:**
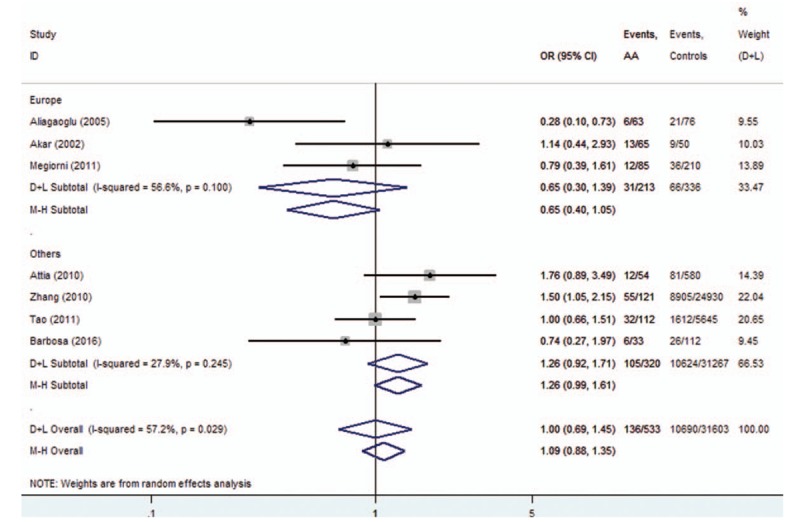
Forest plot of HLA-DRB1∗15 polymorphism and alopecia areata.

**Figure 10:**
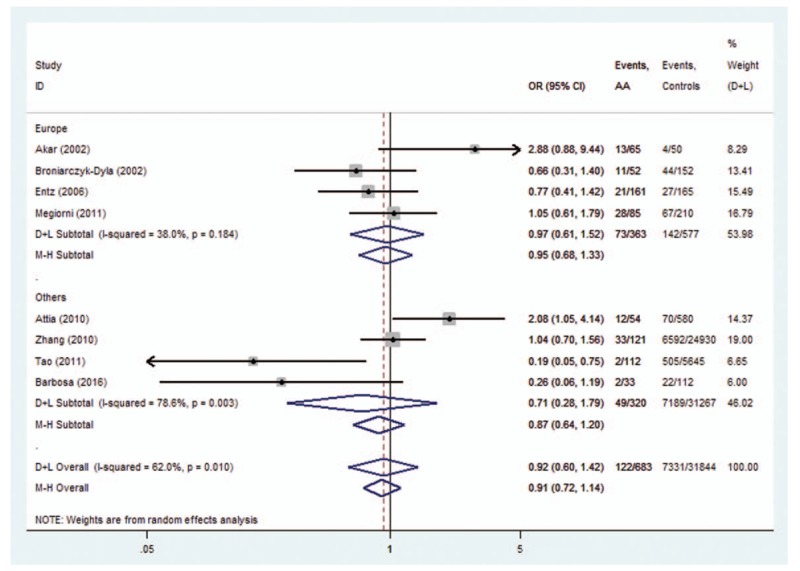
Forest plot of HLA-DRB1∗07 polymorphism and alopecia areata.

**Figure 11:**
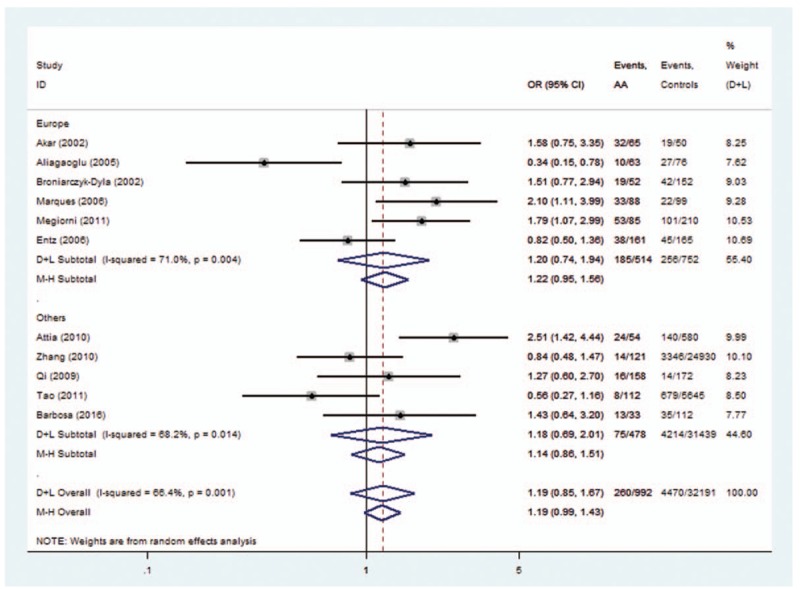
Forest plot of HLA-DRB1∗11 polymorphism and alopecia areata.

The subgroup analysis was conducted on *HLA-DRB1∗03*, *DRB1∗07*, *DRB1∗08*, *DRB1∗11*, and *DRB1∗15* polymorphisms. For *HLA-DRB1∗03* polymorphisms, the analysis of the pooled data of 5 case-control studies revealed low heterogeneity in the Europe subgroup (*P* = .192). A fixed-effects model was used for calculating OR. Overall OR (95% CIs) was 0.40 (0.26–0.60) with *P* < .01 (Fig. 7). For *HLA-DRB1∗08*, a low heterogeneity was observed in the Asia subgroup (*P* = .854). Overall OR (95% CIs) was 0.58 (0.36–0.93) with *P* < .01 (Fig. 8). However, no evidence of association in statistics was found between *HLA-DRB1∗15* (Fig. 9), *HLA-DRB1∗07* (Fig. 10), and *HLA-DRB1∗11* (Fig. 11) polymorphisms and AA in the subgroup analysis.

### 3.4 Sensitivity analyses

**Figure 12:**
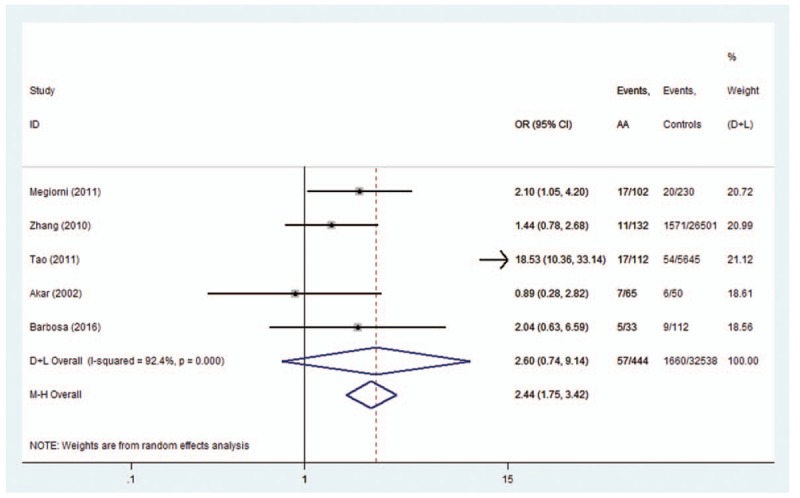
Forest plot of HLA-DRB1∗16^#^ polymorphism and alopecia areata.

A single report involved in the meta-analysis was removed each time to reflect the influence of the individual dataset on the pooled OR. A significant deviation was detected in the study by Tao^[13]^ when analyzing the association between *HLA-DRB1∗16* polymorphisms and AA. After the exclusion of this study, heterogeneity decreased from 92.4% (Fig. 12) to 0% (Fig. 3). The trim-and-fill analysis suggested that no studies (comparisons) were missing from the dataset. It turned out that *HLA-DRB1∗16* polymorphisms conferred a significantly increased risk (Fig. 3).

For others, the corresponding pooled ORs were not materially changed (data not shown), indicating that the results were statistically robust.

### 3.5 Publication bias

Harbord and Eggers tests were not significant in any comparison (*P* > .05, shown in Table 2). The shape of the funnel plot was relatively symmetric for most alleles (Supplemental File 3). They all indicated a low probability of publication bias.

### 3.6 Influence of continent, country, and NOS score

The results of meta-regression analysis showed that continent, country, or NOS score did not account for heterogeneity (Table 3).

**Table 3:**
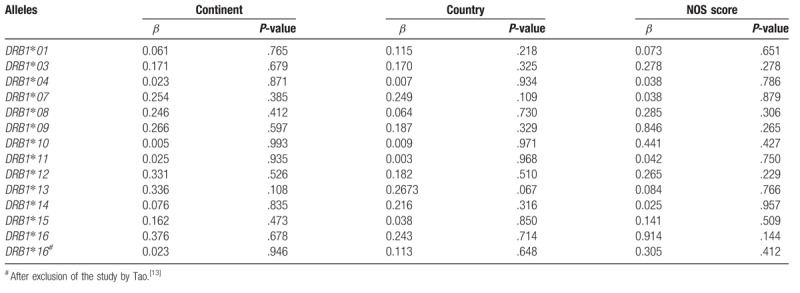
Meta-regression.

## 4 Discussion

A comprehensive evaluation is provided by this systematic review to find out the relationship of *HLA-DRB1* polymorphisms with AA. According to inclusion and exclusion criteria, a total of 1283 cases and 32,343 controls from 12 case-control studies^[12–23]^ were selected and analyzed. The present study revealed that *HLA-DRB1∗04* and *HLA-DRB1∗16* polymorphisms might be associated with increased AA risk, while *HLA-DRB1∗0301*, *HLA-DRB1∗0*9, and *HLA-DRB1∗13* polymorphisms might decrease the AA risk.

The associations between HLA polymorphisms and AA risk have been intensively studied.^[12–23]^ For HLA-*DRB1∗04* polymorphisms, 5 of 8 studies indicated that OR >1 but the 95% CIs cross 1. First, a single study with limited sample size might have been underpowered to clarify the genes with AA risk. Second, a single study can only represent one ethnic background. So after summarizing it quantitatively with a meta-analytic approach, the pooled results indicated that HLA-*DRB1∗04* polymorphisms might be potential risk factors for AA (OR = 1.49, Fig. 2). A similar situation occurs when analyzing the association of *HLA-DRB1∗09, HLA-DRB1∗16*, and *HLA-DRB1∗13* polymorphisms with AA. The purpose of this study was to increase the statistical power, evaluate the evidence from studies by summarizing it quantitatively with a meta-analytic approach, and get a reliable conclusion.

One genome-wide meta-analysis discussed the relationship between HLA and AA.^[1]^ The study indicated *HLA-DRB1∗04:01* polymorphisms as the potential risk factors for AA (OR = 1.64). It included 2489 cases and 5287 controls from the United States and Central Europe. Many studies implied that ethnic difference might be associated with the genotype distribution. Besides the United States and Europe, the present study included Asian and African countries. The results revealed that *HLA-DRB1∗04* polymorphisms might be a risk factor for AA, which is a supplement of the previous meta-analyses.

Heterogeneity could potentially impact the results of all meta-analyses.^[3]^ In our research, statistical heterogeneity was noticed among some analyses. We therefore explored the sources of heterogeneity to examine whether the results were robust. First, we have conducted meta-regression analysis to reveal whether continent, country, or NOS score could lead to heterogeneity. However, meta-regression indicated that these covariates were not statistically significant (*P* > .05). Second, sensitivity analyses were performed. It indicated that after the exclusion of the study by Tao,^[13]^ heterogeneity decreased from 92.4% (Fig. 12) to 0% (Fig. 3) when studying the association of *HLA*-*DRB1∗16* polymorphisms with AA. Additionally, subgroup analyses revealed that geographical factors might have led to the heterogeneity when studying the association of *HLA*-*DRB1∗03* and *HLA*-*DRB1∗08* polymorphisms with AA. However, because of the limited studies included in the subgroup analyses, further studies and analyses are needed to validate the findings.

To avoid local literature bias,^[33]^ we obtained and included both English and Chinese language reports. And yet, some shortcomings of the analysis could not be neglected. First, the number of included studies was limited because the incidence of *HLA-DRB1* genotypes was low. Enough information could not be obtained on clinical type and magnitude for subgroup analysis due to the limited number of included studies. Second, it was uncertain whether the cases were comparably representative, although significant publication bias between studies was not detected.

## 5 Conclusion

The present study revealed that *HLA-DRB1∗04* and *HLA-DRB1∗16* polymorphisms might be associated with increased AA risk, while *HLA-DRB1∗0301*, *HLA-DRB1∗09, HLA-DRB1∗13* polymorphisms might decrease the AA risk. Studies with adequate methodological quality on gene–gene and gene–environment interactions are needed to validate the results in the future.

## Acknowledgments

The authors sincerely thank all the authors of the original articles.

## Author contributions

**Funding acquisition:** Conghua Ji, Shan Liu.

**Methodology:** Conghua Ji, Shan Liu.

**Project administration:** Conghua Ji, Shan Liu.

**Writing – original draft:** Conghua Ji.

**Resources:** Shan Liu, Kan Zhu, Qing Chen.

**Supervision:** Shan Liu, Yi Cao.

**Validation:** Shan Liu, Kan Zhu, Qing Chen.

**Writing – review and editing:** Shan Liu, Yi Cao.

**Investigation:** Kan Zhu, Hongbin Luo, Qiushuang Li.

**Data curation:** Hongbin Luo, Qiushuang Li.

**Formal analysis:** Hongbin Luo, Ying Zhang.

**Software:** Ying Zhang, Sijia Huang.

**Visualization:** Sijia Huang.

**Conceptualization:** Yi Cao.

## Supplementary Material

Supplemental Digital Content

## References

[1] Betz RC Petukhova L Ripke S Genome-wide meta-analysis in alopecia areata resolves HLA associations and reveals two new susceptibility loci. Nat Commun 2015;6:5966. 25608926 10.1038/ncomms6966 PMC4451186

[2] Olsen EA Hordinsky MK Price VH Alopecia areata investigational assessment guidelines–Part II. National Alopecia Areata Foundation. J Am Acad Dermatol 2004;51:440–7. 15337988 10.1016/j.jaad.2003.09.032

[3] Liu S Li Q Zhang Y Association of Human Leukocyte Antigen DRB1∗15 and DRB1∗15:01 polymorphisms with response to immunosuppressive therapy in patients with aplastic anemia: a meta-analysis. PLoS One 2016;11:e0162382. 27611583 10.1371/journal.pone.0162382 PMC5017877

[4] Niu Z Zhang P Tong Y Value of HLA-DR genotype in systemic lupus erythematosus and lupus nephritis: a meta-analysis. Int J Rheum Dis 2015;18:17–28. 25546242 10.1111/1756-185X.12528

[5] Shi T Lv W Zhang L Association of HLA-DR4/HLA-DRB1∗04 with Vogt-Koyanagi-Harada disease: a systematic review and meta-analysis. Sci Rep 2014;4:6887. 25382027 10.1038/srep06887 PMC4225552

[6] Xiao D Ye X Zhang N A meta-analysis of interaction between Epstein-Barr virus and HLA-DRB1∗1501 on risk of multiple sclerosis. Sci Rep 2015;5:18083. 26656273 10.1038/srep18083 PMC4676020

[7] Islam N Leung PSC Huntley AC The autoimmune basis of alopecia areata: a comprehensive review. Autoimmun Rev 2015;14:81–9. 25315746 10.1016/j.autrev.2014.10.014

[8] Brocker EB Echternacht-Happle K Hamm H Abnormal expression of class I and class II major histocompatibility antigens in alopecia areata: modulation by topical immunotherapy. J Invest Dermatol 1987;88:564–8. 3471816 10.1111/1523-1747.ep12470166

[9] Messenger AG Bleehen SS Expression of HLA-DR by anagen hair follicles in alopecia areata. J Invest Dermatol 1985;85:569–72. 2415641 10.1111/1523-1747.ep12277414

[10] Alzolibani AA Epidemiologic and genetic characteristics of alopecia areata (part 1). Acta Dermatovenerol Alp Pannonica Adriat 2011;20:191–8. 22367375

[11] Golan D Lander ES Rosset S Measuring missing heritability: inferring the contribution of common variants. Proc Natl Acad Sci USA 2014;111:E5272–81. 25422463 10.1073/pnas.1419064111 PMC4267399

[12] Entz P Blaumeiser B Betz RC Investigation of the HLA-DRB1 locus in alopecia areata. Eur J Dermatol 2006;16:363–7. 16935791

[13] Tao H. Association of severe Alopecia Areata and HLA-DRB1 alleles in Southern Chinese Han. Guangzhou Medical Colleage; 2011.

[14] Qi S. Association of HLA-DRBl∗03, ∗04 and ∗11 alleles with alopecia areata in Han people in Eastern China. Fudan University; 2009.

[15] Akar A Orkunoglu E Sengul A HLA class II alleles in patients with alopecia areata. Eur J Dermatol 2002;12:236–9. 11978563

[16] Attia EA El Shennawy D Sefin A Serum Interleukin-4 and total immunoglobulin E in nonatopic alopecia areata patients and HLA-DRB1 typing. Dermatol Res Pract 2010;2010:503587. 20671941 10.1155/2010/503587 PMC2910459

[17] Broniarczyk-Dyla G Prusinska-Bratos M Dubla-Berner M The protective role of the HLA-DR locus in patients with various clinical types of alopecia areata. Arch Immunol Therap Exp 2002;50:333–6. 12455867

[18] Megiorni F Pizzuti A Mora B Genetic association of HLA-DQB1 and HLA-DRB1 polymorphisms with alopecia areata in the Italian population. Br J Dermatol 2011;165:823–7. 21692766 10.1111/j.1365-2133.2011.10466.x

[19] Zhang B Wu Y Cheng S Polymorphism analysis of HLA-A,B and DRB1 in patients with alopecia areata. Int J Blood Transfus Hematol 2010;33:289–92.

[20] Barbosa AM Prestes-Carneiro LE Sobral AR Lack of association between alopecia areata and HLA class I and II in a southeastern Brazilian population. An Bras Dermatol 2016;91:284–9. 27438193 10.1590/abd1806-4841.20164250 PMC4938270

[21] Aliagaoglu C Pirim I Atasoy M Association between alopecia areata and HLA class I and II in Turkey. J Dermatol 2005;32:711–4. 16361713 10.1111/j.1346-8138.2005.tb00830.x

[22] Barahmani N de Andrade M Slusser JP Human leukocyte antigen class II alleles are associated with risk of alopecia areata. J Invest Dermatol 2008;128:240–3. 17637820 10.1038/sj.jid.5700973

[23] Marques Da Costa C Dupont E Van Der Cruys M Earlier occurrence of severe alopecia areata in HLA-DRB1∗11-positive patients. Dermatology 2006;213:12–4. 16778420 10.1159/000092831

[24] Knobloch K Yoon U Vogt PM Preferred reporting items for systematic reviews and meta-analyses (PRISMA) statement and publication bias. J Craniomaxillofac Surg 2011;39:91–2. 21145753 10.1016/j.jcms.2010.11.001

[25] Bero L Rennie D The Cochrane Collaboration. Preparing, maintaining, and disseminating systematic reviews of the effects of health care. JAMA 1995;274:1935–8. 8568988 10.1001/jama.274.24.1935

[26] Higgins JP Thompson SG Deeks JJ Measuring inconsistency in meta-analyses. BMJ 2003;327:557–60. 12958120 10.1136/bmj.327.7414.557 PMC192859

[27] DerSimonian R Laird N Meta-analysis in clinical trials. Control Clin Trials 1986;7:177–88. 3802833 10.1016/0197-2456(86)90046-2

[28] Mantel N Haenszel W Statistical aspects of the analysis of data from retrospective studies of disease. J Natl Cancer Inst 1959;22:719–48. 13655060

[29] Patsopoulos NA Evangelou E Ioannidis JP Sensitivity of between-study heterogeneity in meta-analysis: proposed metrics and empirical evaluation. Int J Epidemiol 2008;37:1148–57. 18424475 10.1093/ije/dyn065 PMC6281381

[30] Harbord RM Harris RJ Sterne JAC Updated tests for small-study effects in meta-analyses. Stata J 2009;9:197–210.

[31] Egger M Davey Smith G Schneider M Bias in meta-analysis detected by a simple, graphical test. BMJ 1997;315:629–34. 9310563 10.1136/bmj.315.7109.629 PMC2127453

[32] Weinhandl ED Duval S Generalization of trim and fill for application in meta-regression. Res Synth Methods 2012;3:51–67. 26061999 10.1002/jrsm.1042

[33] Pan Z Trikalinos TA Kavvoura FK Local literature bias in genetic epidemiology: an empirical evaluation of the Chinese literature. PLoS Med 2005;2:e334. 16285839 10.1371/journal.pmed.0020334 PMC1285066

