# Supplementary material for: HLA-DRB1 polymorphisms and alopecia areata disease risk: A systematic review and meta-analysis
Source: Medicine (Baltimore). 2018 Aug 10;97(32):e11790. doi: 10.1097/MD.0000000000011790 (PMC6133534; doi:10.1097/MD.0000000000011790)
Supplement: Supplemental Digital Content [file medi-97-e11790-s001.pdf]

PROSPERO International prospective register of systematic reviews

Review title and timescale

1 Review title

Give the working title of the review. This must be in English. Ideally it should state succinctly the interventions or exposures being reviewed and the associated health or social problem being addressed in the review.

HLA- DRB1 polymorphism and alopecia areata: evidence from a meta-analysis

2 Original language title

For reviews in languages other than English, this field should be used to enter the title in the language of the review. This will be displayed together with the English language title.

3 Anticipated or actual start date

Give the date when the systematic review commenced, or is expected to commence.

01/05/2015

4 Anticipated completion date

Give the date by which the review is expected to be completed.

31/07/2015

5 Stage of review at time of this submission

Indicate the stage of progress of the review by ticking the relevant boxes. Reviews that have progressed beyond the point of completing data extraction at the time of initial registration are not eligible for inclusion in PROSPERO. This field should be updated when any amendments are made to a published record.

The review has not yet started ×

Review stage	Started	Completed
Preliminary searches	Yes	Yes
Piloting of the study selection process	Yes	Yes
Formal screening of search results against eligibility criteria	Yes	Yes
Data extraction	Yes	Yes
Risk of bias (quality) assessment	Yes	Yes
Data analysis	Yes	Yes

Provide any other relevant information about the stage of the review here.

Review team details

6 Named contact

The named contact acts as the guarantor for the accuracy of the information presented in the register record.

Dr Liu

7 Named contact email

Enter the electronic mail address of the named contact.

graystar92@163.com

8 Named contact address

Enter the full postal address for the named contact.

54 Youdian Road, Hangzhou. Zhejiang Province. P. R. China.

9 Named contact phone number

Enter the telephone number for the named contact, including international dialing code.

86-18806715135

10 Organisational affiliation of the review

Full title of the organisational affiliations for this review, and website address if available. This field may be completed as 'None' if the review is not affiliated to any organisation.

none

Website address:

11 Review team members and their organisational affiliations

Give the title, first name and last name of all members of the team working directly on the review. Give the organisational affiliations of each member of the review team.

Title	First name	Last name	Affiliation
Dr	shan	liu	
Professor	conghua	ji	

12 Funding sources/sponsors

Give details of the individuals, organizations, groups or other legal entities who take responsibility for initiating, managing, sponsoring and/or financing the review. Any unique identification numbers assigned to the review by the individuals or bodies listed should be included.

None

13 Conflicts of interest

List any conditions that could lead to actual or perceived undue influence on judgements concerning the main topic investigated in the review.

Are there any actual or potential conflicts of interest?

None known

14 Collaborators

Give the name, affiliation and role of any individuals or organisations who are working on the review but who are not listed as review team members.

Title	First name	Last name	Organisation details

Review methods

15 Review question(s)

State the question(s) to be addressed / review objectives. Please complete a separate box for each question.

We undertook this study to review and quantitatively analyze the association between human leukocyte antigen (HLA) DRB1 polymorphisms and susceptibility fo alopecia areata (AA).

16 Searches

Give details of the sources to be searched, and any restrictions (e.g. language or publication period). The full search strategy is not required, but may be supplied as a link or attachment.

Several databases (MEDLINE/PubMed, Chinese China National Knowledge Infrastructure CNKI, EMBASE, Web of Science, Cochrane databases) were searched through June 2015 for all publications on the association between HLA Polymorphism and AA. The search terms were as follows: (‘‘Alopecia areata’’) and(‘‘HLA’’ or ‘‘human leukocyte antigen’’) and(‘‘polymorphism’’ or ‘‘variant’’ or ‘‘genotype’’). No language limitations were used. In addition, we also searched references of retrieved articles.

17 URL to search strategy

If you have one, give the link to your search strategy here. Alternatively you can e-mail this to PROSPERO and we will store and link to it.

I give permission for this file to be made publicly available

Yes

18 Condition or domain being studied

Give a short description of the disease, condition or healthcare domain being studied. This could include health and wellbeing outcomes.

Papers involving study of HLA expression in alopecia areata (AA).

19 Participants/population

Give summary criteria for the participants or populations being studied by the review. The preferred format includes details of both inclusion and exclusion criteria.

Diagnosis of a alopecia areata disease in the patient study group, and controls should be alopecia areata-free subjects

20 Intervention(s), exposure(s)

Give full and clear descriptions of the nature of the interventions or the exposures to be reviewed

Case-control or cohort studies related to the association of HLA-DRB1 polymorphism and alopecia areata risk.

21 Comparator(s)/control

Where relevant, give details of the alternatives against which the main subject/topic of the review will be compared (e.g. another intervention or a non-exposed control group).

Alopecia areata-free patients or healthy persons as controls.

22 Types of study to be included initially

Give details of the study designs to be included in the review. If there are no restrictions on the types of study design eligible for inclusion, this should be stated.

In the present report we included cases-control or cohort studies which evaluate the association between HLA-DRB1 with alopecia areata.

23 Context

Give summary details of the setting and other relevant characteristics which help define the inclusion or exclusion criteria.

24 Primary outcome(s)

Give the most important outcomes.

HLA-DRB1 polymorphism is associated with increased alopecia areata risk.

Give information on timing and effect measures, as appropriate.

25 Secondary outcomes

List any additional outcomes that will be addressed. If there are no secondary outcomes enter None.

None

Give information on timing and effect measures, as appropriate.

26 Data extraction, (selection and coding)

Give the procedure for selecting studies for the review and extracting data, including the number of researchers involved and how discrepancies will be resolved. List the data to be extracted.

Data extraction was performed independently by two investigators according to the inclusion criteria listed above.The third participant was consulted for discussion to reach agreement concerning discrepancies. The following items were extracted from each study: first author’s last name, publication date, country of origin, the Newcastle-Ottawa Scale (NOS), numbers of cases and controls, genotyping method.

27 Risk of bias (quality) assessment

State whether and how risk of bias will be assessed, how the quality of individual studies will be assessed, and whether and how this will influence the planned synthesis.

Quality of the included articles was evaluated using Newcastle-Ottawa Scale (NOS) scale. The NOS contains eight items categorized into three dimensions including selection, comparability, and exposure. For each item a series of response options is provided.A star system is used to allow a semi-quantitative assessment of study quality, such that the highest quality studies are awarded a maximum of one star for each item with the exception of the item related to comparability that allows the assignment of two stars. The NOS ranges between zero and nine stars.

28 Strategy for data synthesis

Give the planned general approach to be used, for example whether the data to be used will be aggregate or at the level of individual participants, and whether a quantitative or narrative (descriptive) synthesis is planned. Where appropriate a brief outline of analytic approach should be given.

Review manager 5.3 software was used for statistical analysis toperform meta-analysis. Heterogeneity was checked by the Chi2 test (Cochran 1954) and the I-squared statistic (Higgins 2003). The criteria for identification of heterogeneity was a P value less than 0.10 for the Chi-squared test and an I-squared statistic greater than 50%. When there was no statistical evidence for heterogeneity in effect sizes, we used the fixed-effect model (Mantel 1959). When significant heterogeneity was identified, we used the random-effects model (DerSimonian 1986) and

explored sources of significant heterogeneity.

29 Analysis of subgroups or subsets
Give any planned exploration of subgroups or subsets within the review. ‘None planned’ is a valid response if no subgroup analyses are planned.
Subgroup analyses were performed by ethnicity.

Review general information

30 Type of review
Select the type of review from the drop down list.
Diagnostic, Epidemiologic, Prognostic

31 Language
Select the language(s) in which the review is being written and will be made available, from the drop down list. Use the control key to select more than one language.
English

Will a summary/abstract be made available in English?
Yes

32 Country
Select the country in which the review is being carried out from the drop down list. For multi-national collaborations select all the countries involved. Use the control key to select more than one country.
China

33 Other registration details
Give the name of any organisation where the systematic review title or protocol is registered together with any unique identification number assigned. If extracted data will be stored and made available through a repository such as the Systematic Review Data Repository (SRDR), details and a link should be included here.

34 Reference and/or URL for published protocol
Give the citation for the published protocol, if there is one.
Give the link to the published protocol, if there is one. This may be to an external site or to a protocol deposited with CRD in pdf format.

I give permission for this file to be made publicly available
Yes

35 Dissemination plans
Give brief details of plans for communicating essential messages from the review to the appropriate audiences.
Do you intend to publish the review on completion?
Yes

36 Keywords
Give words or phrases that best describe the review. (One word per box, create a new box for each term)

37 Details of any existing review of the same topic by the same authors
Give details of earlier versions of the systematic review if an update of an existing review is being registered, including full bibliographic reference if possible.

38 Current review status
Review status should be updated when the review is completed and when it is published.
Ongoing

39 Any additional information

Provide any further information the review team consider relevant to the registration of the review.

40 Details of final report/publication(s)

This field should be left empty until details of the completed review are available.
Give the full citation for the final report or publication of the systematic review.
Give the URL where available.

Supplemental File 2_Fig1. Forest plot of HLA-DRB1*01 polymorphism and alopecia areata.

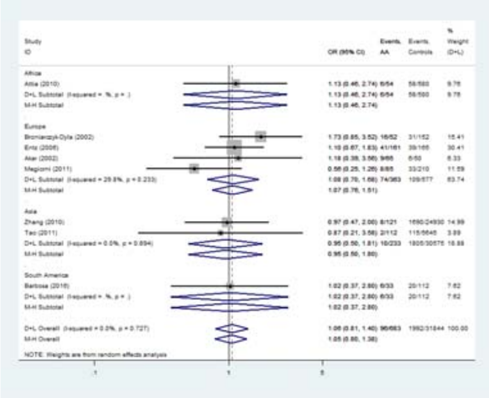

Supplemental File 2_Fig2. Forest plot of HLA-DRB1*10 polymorphism and alopecia areata.

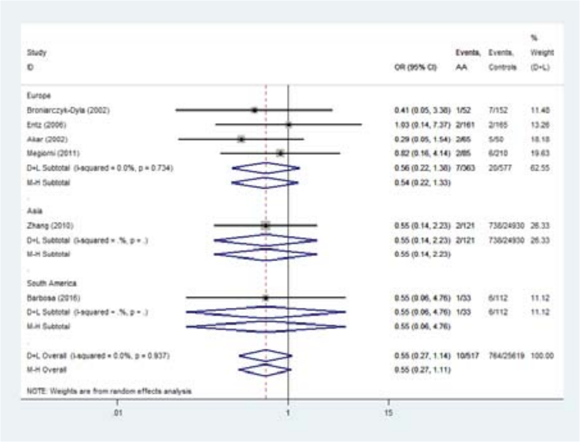

Supplemental File 2_Fig3. Forest plot of HLA-DRB1*12 polymorphism and alopecia areata.

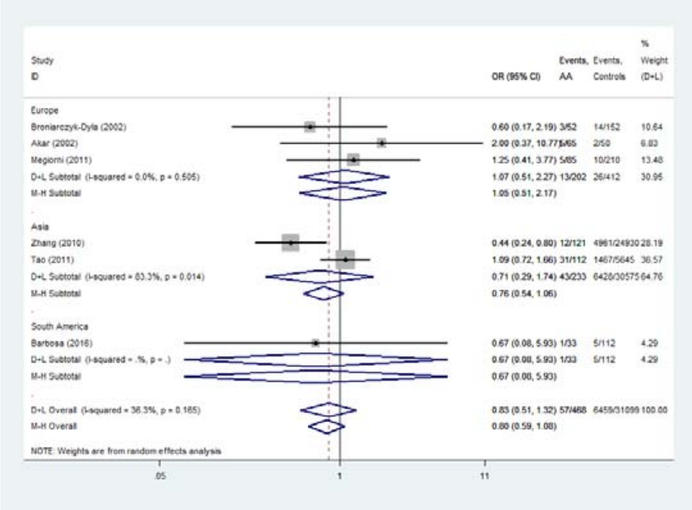

Supplemental File 2_Fig4. Forest plot of HLA-DRB1*14 polymorphism and alopecia areata.

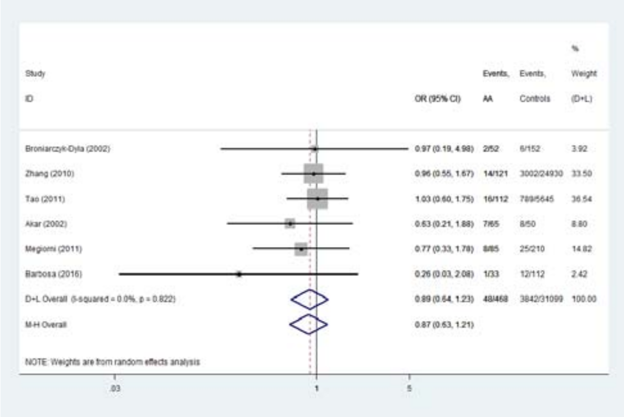

Supplemental File 2_Fig5. Forest plot of HLA-DRB1*1516 polymorphism and alopecia areata.

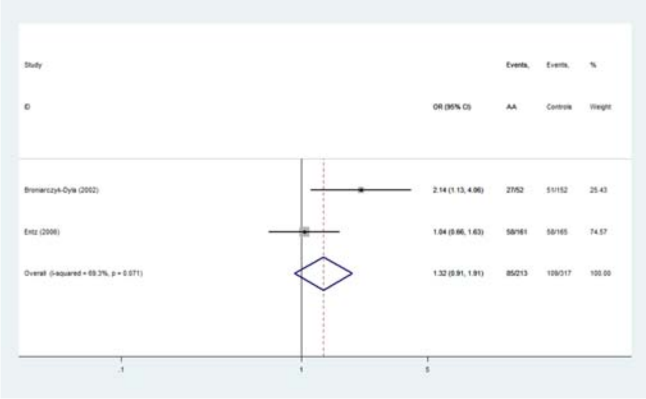

Supplemental File 3_Fig1. Funnel plot of HLA-DRB1*01 polymorphism and alopecia areata.

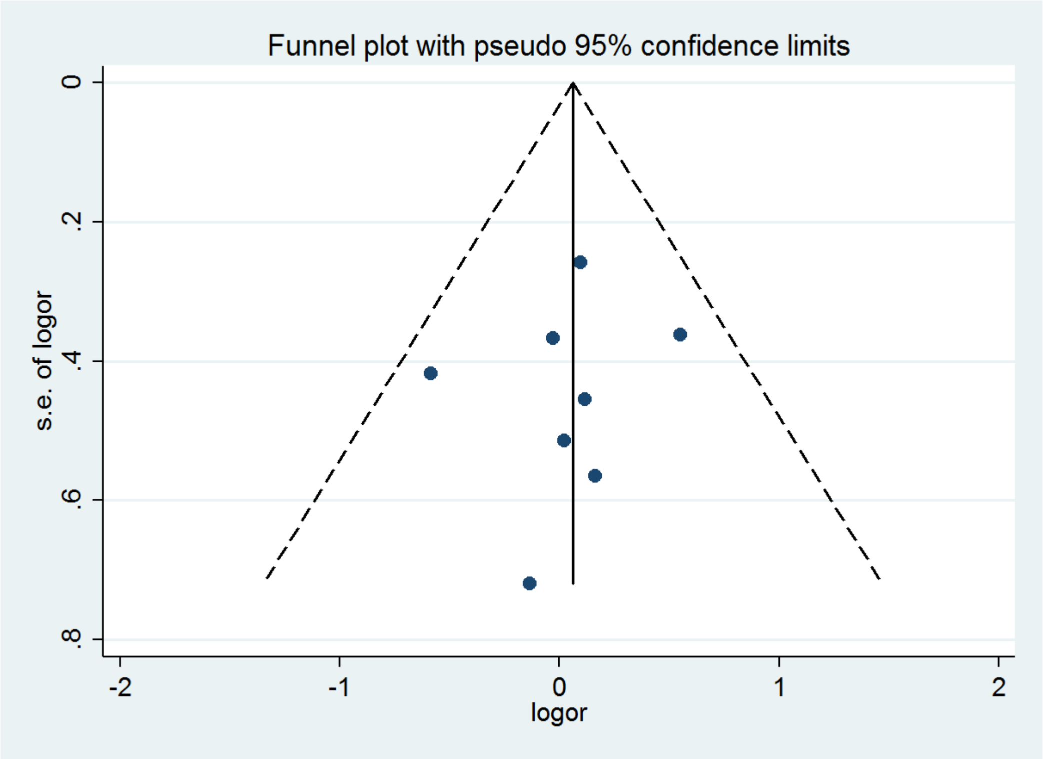

Supplemental File 3_Fig2. Funnel plot of HLA-DRB1*03 polymorphism and alopecia areata.

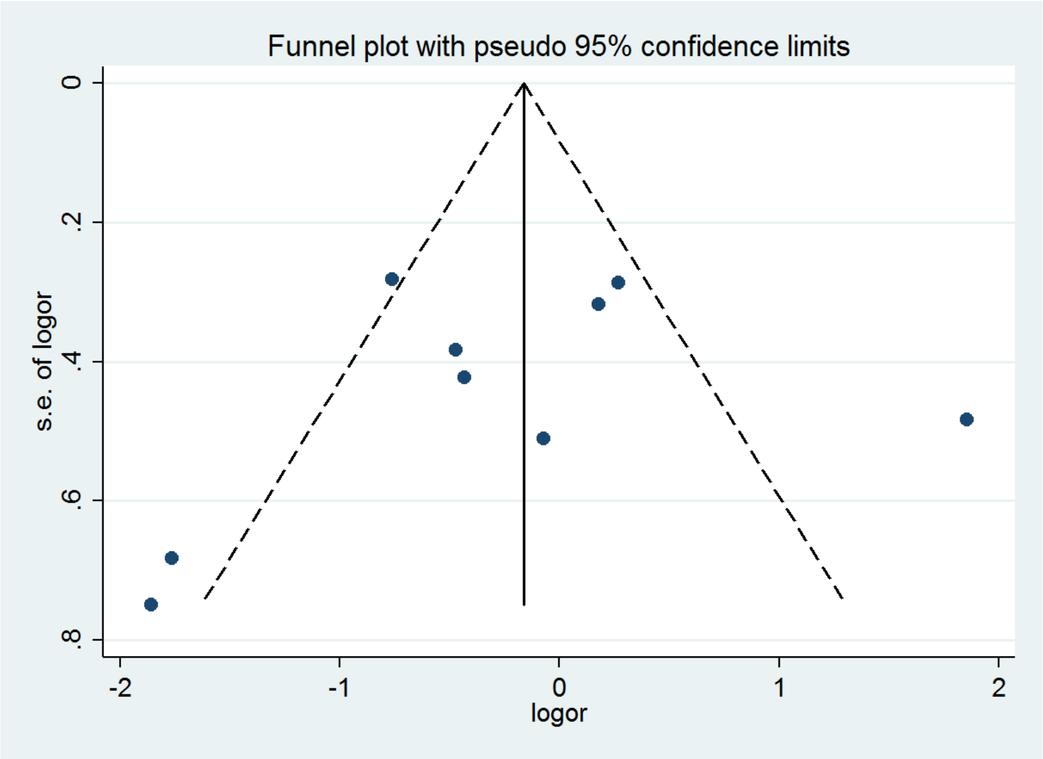

Supplemental File 3_Fig3. Funnel plot of HLA-DRB1*04 polymorphism and alopecia areata.

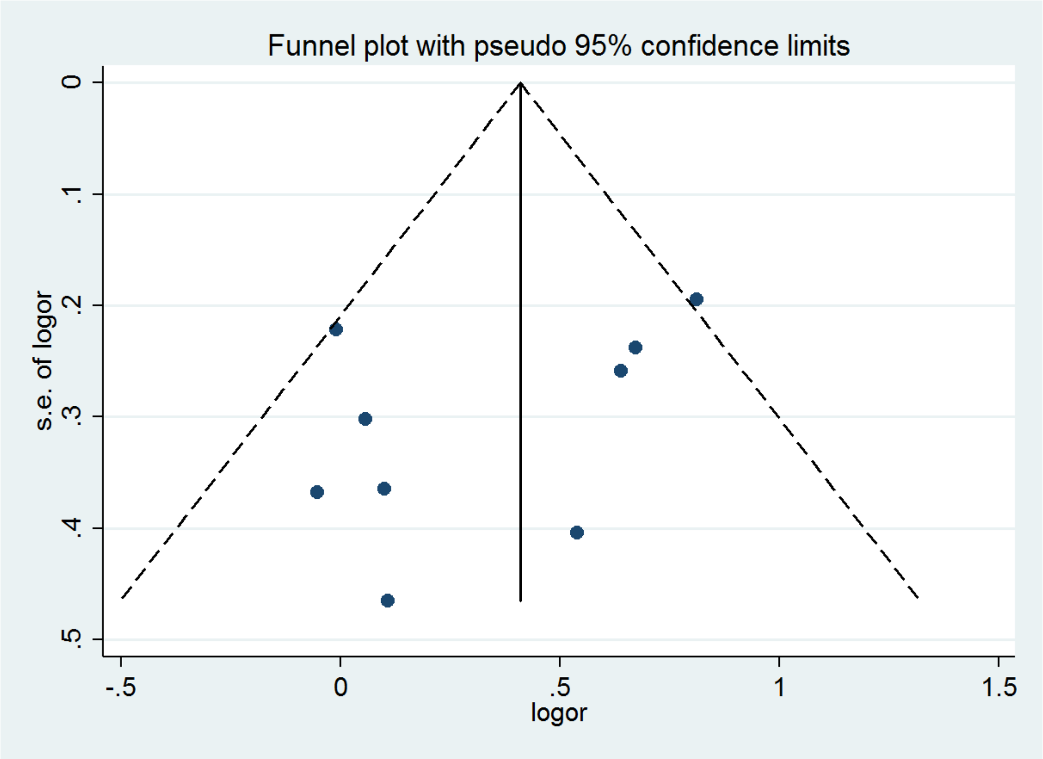

Supplemental File 3_Fig4. Funnel plot of HLA-DRB1*07 polymorphism and alopecia areata.

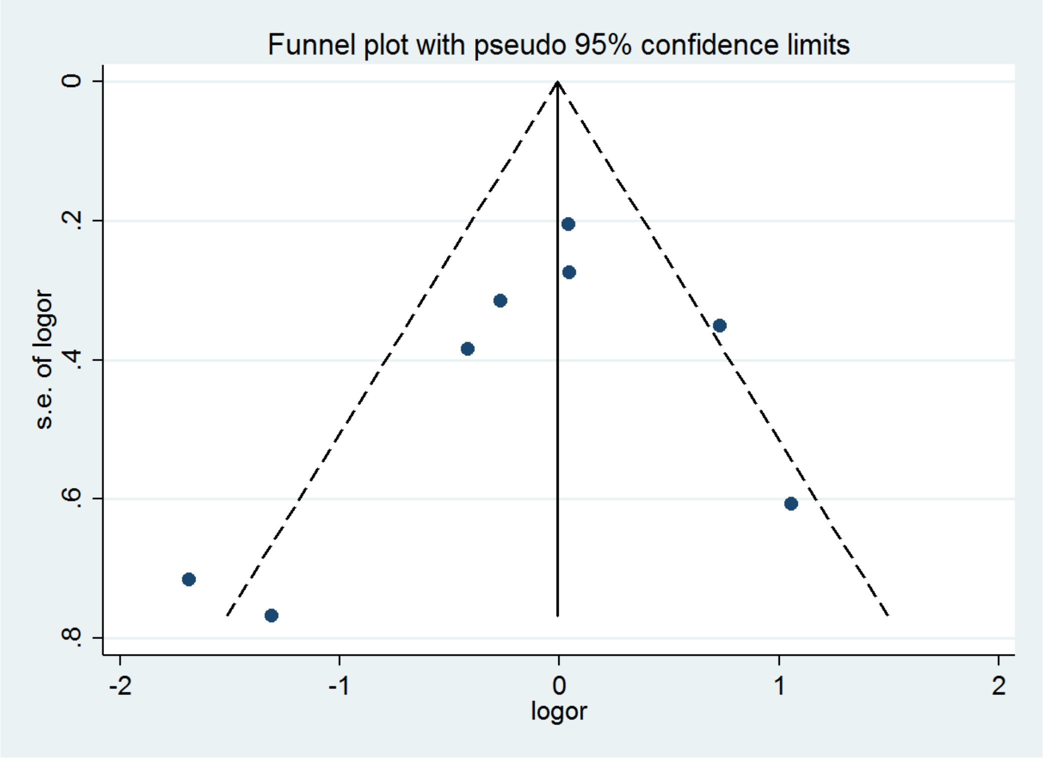

Supplemental File 3_Fig5. Funnel plot of HLA-DRB1*08 polymorphism and alopecia areata.

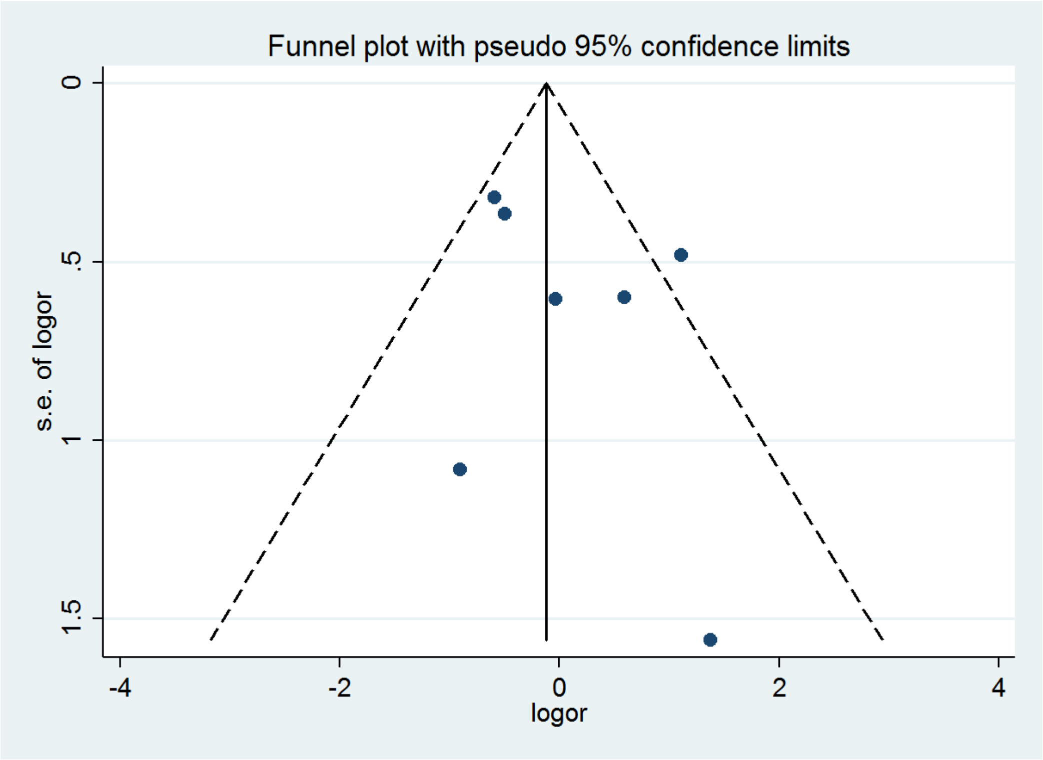

Supplemental File 3_Fig6. Funnel plot of HLA-DRB1*09 polymorphism and alopecia areata.

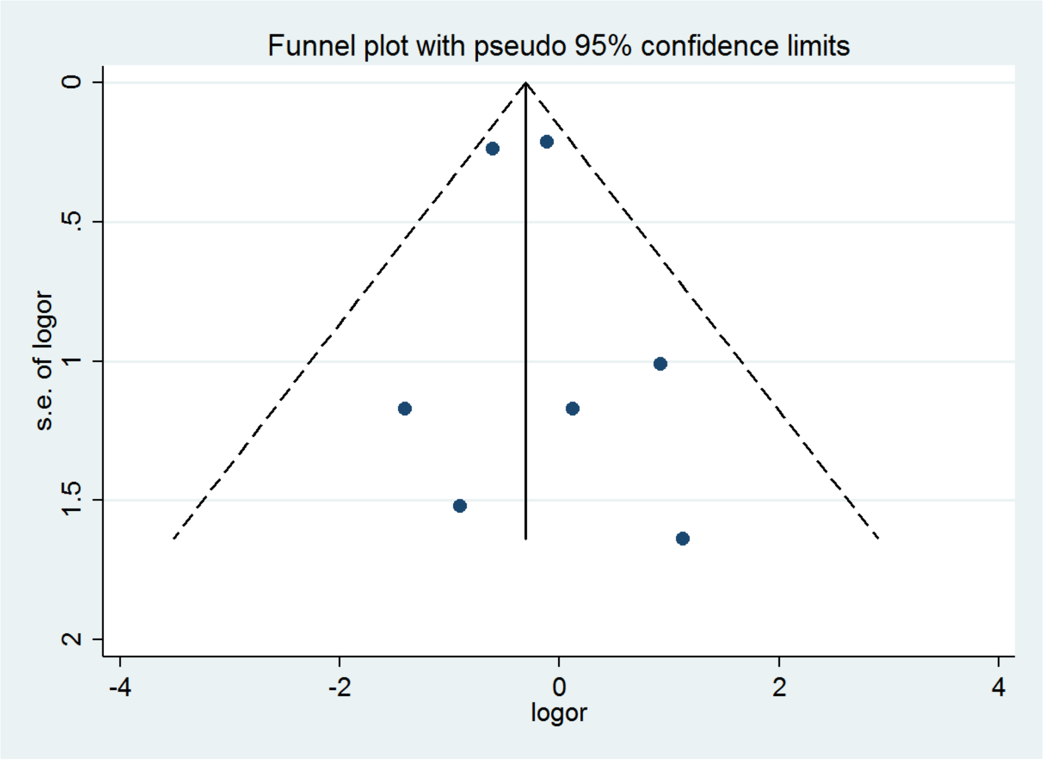

Supplemental File 3_Fig7. Funnel plot of HLA-DRB1*10 polymorphism and alopecia areata.

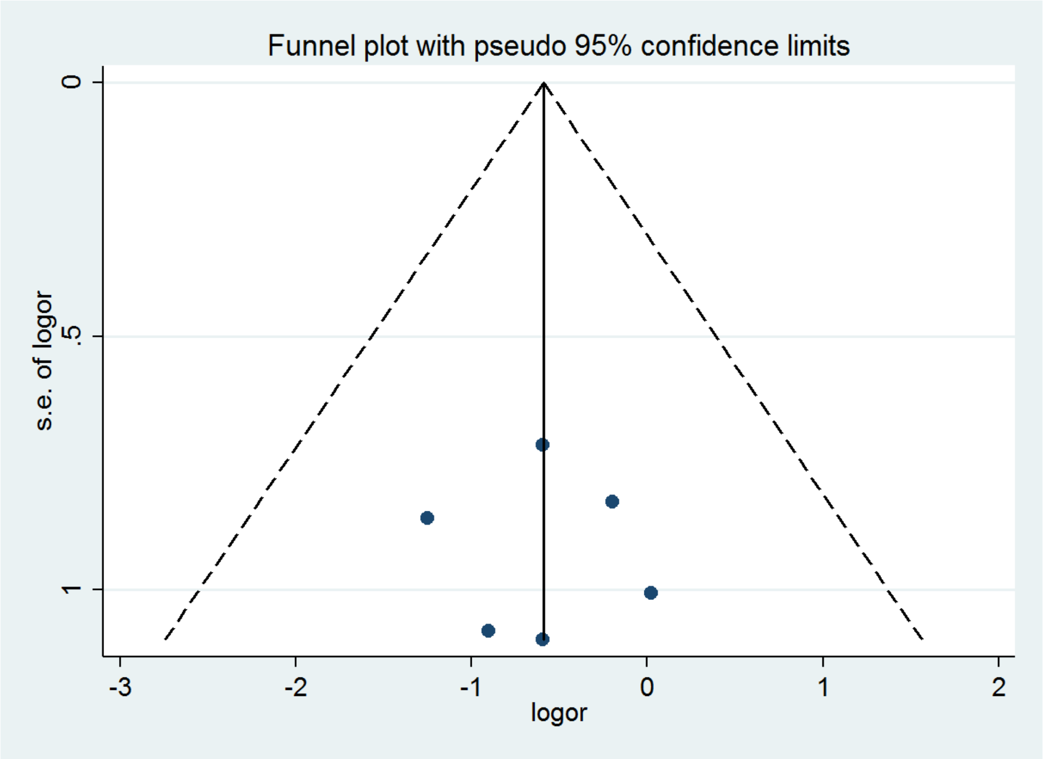

Supplemental File 3_Fig8. Funnel plot of HLA-DRB1*11 polymorphism and alopecia areata.

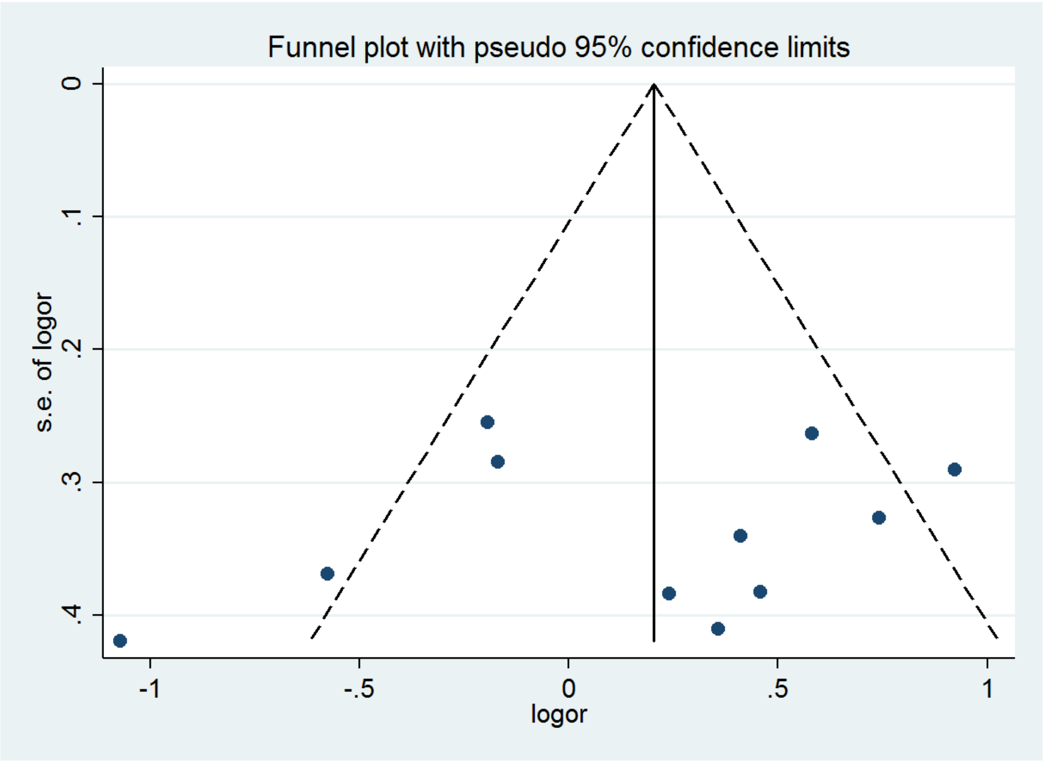

Supplemental File 3_Fig9. Funnel plot of HLA-DRB1*12 polymorphism and alopecia areata.

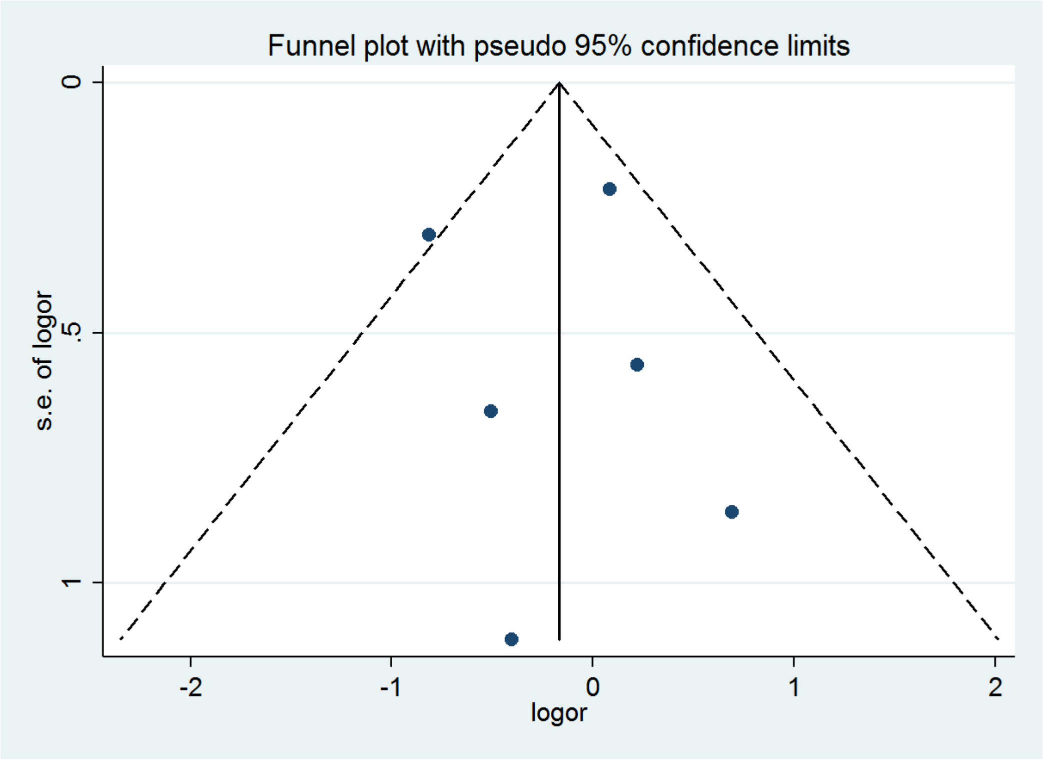

Supplemental File 3_Fig10. Funnel plot of HLA-DRB1*13 polymorphism and alopecia areata.

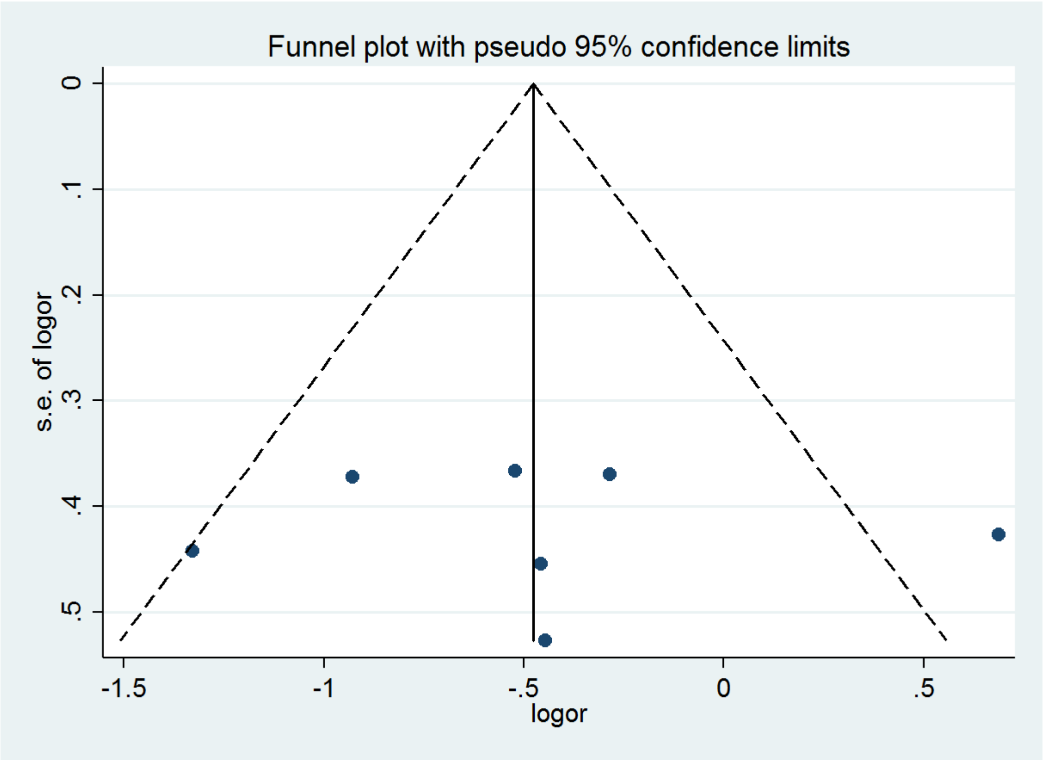

Supplemental File 3_Fig11. Funnel plot of HLA-DRB1*14 polymorphism and alopecia areata.

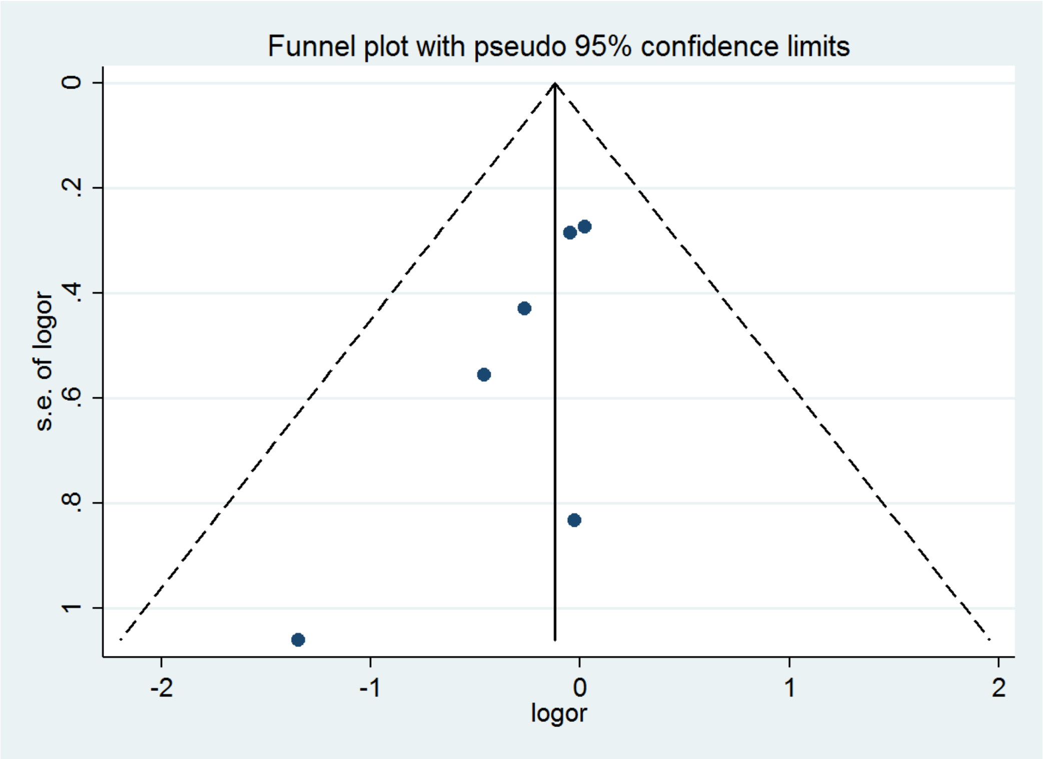

Supplemental File 3_Fig12. Funnel plot of HLA-DRB1*15 polymorphism and alopecia areata.

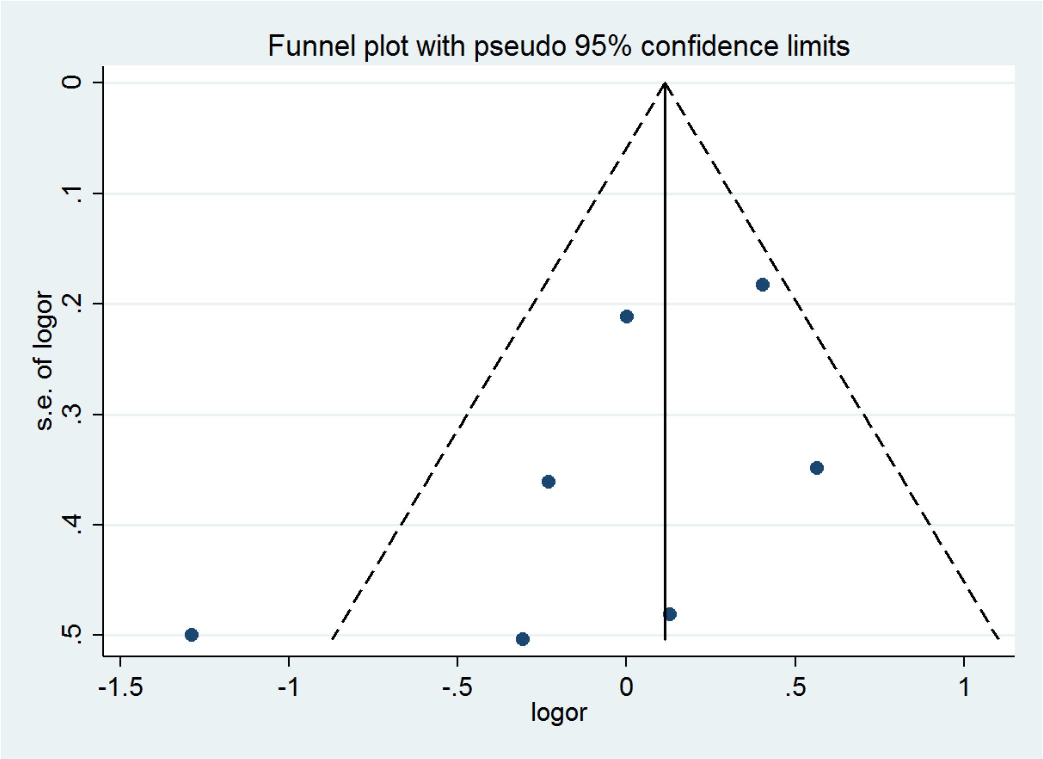

Supplemental File 3_Fig13. Funnel plot of HLA-DRB1*16 polymorphism and alopecia areata.

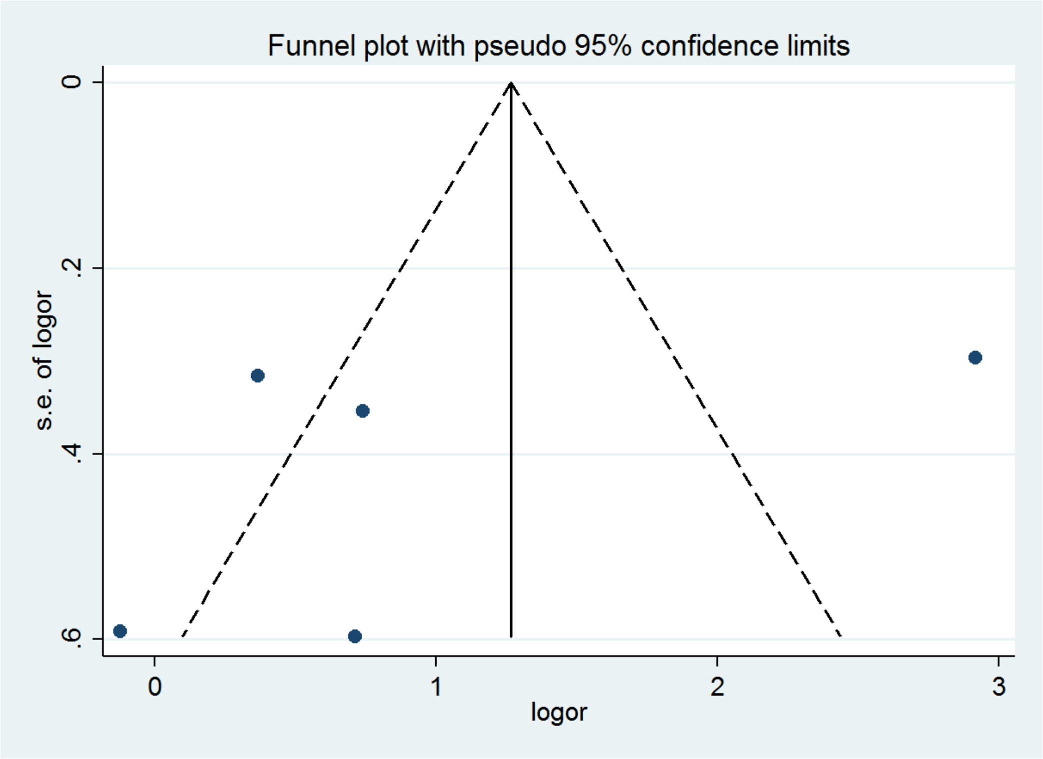

Section/topic	#	Checklist item

TITLE

Title	1	Identify the report as a systematic review, meta-analysis, or both.
[redacted]		

ABSTRACT

[redacted]

Structured summary	2	Provide a structured summary including, as applicable: background; objective participants, and interventions; study appraisal and synthesis methods; result implications of key findings; systematic review registration number.
[redacted]	[redacted]	[redacted]

INTRODUCTION

Rationale	3	Describe the rationale for the review in the context of what is already known.
[redacted]		

Objectives	4	Provide an explicit statement of questions being addressed with reference to outcomes, and study design (PICOS).
[redacted]		[redacted]

METHODS

[redacted]

Protocol and registration	5	Indicate if a review protocol exists, if and where it can be accessed (e.g., Wel registration information including registration number.
[redacted]		[redacted]

Eligibility criteria	6	Specify study characteristics (e.g., PICOS, length of follow-up) and report cha language, publication status) used as criteria for eligibility, giving rationale.
[redacted]		[redacted]

Information sources	7	Describe all information sources (e.g., databases with dates of coverage, con additional studies) in the search and date last searched.
[redacted]	[redacted]	[redacted]

Search	8	Present full electronic search strategy for at least one database, including any repeated.
[redacted]		[redacted]

Study selection	9	State the process for selecting studies (i.e., screening, eligibility, included in s[illegible] included in the meta-analysis).
[redacted]		[redacted]

Data collection process	10	Describe method of data extraction from reports (e.g., piloted forms, indepen for obtaining and confirming data from investigators.
[redacted]		[redacted]

Data items	11	List and define all variables for which data were sought (e.g., PICOS, funding simplifications made.
[redacted]		[redacted]

Risk of bias in individual studies	12	Describe methods used for assessing risk of bias of individual studies (includ at the study or outcome level), and how this information is to be used in any c
[redacted]		

Summary measures	13	State the principal summary measures (e.g., risk ratio, difference in means).
[redacted]		

Synthesis of results	14	Describe the methods of handling data and combining results of studies, if do<br>(e.g., I^2) for each meta-analysis.
[redacted]		[redacted]

Page 1 of 2

[redacted]

#	Checklist item

s studies	15	Specify any assessment of risk of bias that may affect the cumulative evidence (e.g., publication bias, selective reporting within studies).
[redacted]		[redacted]

es	16	Describe methods of additional analyses (e.g., sensitivity or subgroup analyses, meta-regression), if done, indicatin which were pre-specified.	
[redacted]	[redacted]	[redacted]	

[redacted]

	17	Give numbers of studies screened, assessed for eligibility, and included in the review, with reasons for exclusions a each stage, ideally with a flow diagram.	
[redacted]		[redacted]	

tics	18	For each study, present characteristics for which data were extracted (e.g., study size, PICOS, follow-up period) an provide the citations.
[redacted]		[redacted]

[illegible] studies	19	Present data on risk of bias of each study and, if available, any outcome level assessment (see item 12).
[redacted]		

ual studies	20	For all outcomes considered (benefits or harms), present, for each study: (a) simple summary data for each intervention group (b) effect estimates and confidence intervals, ideally with a forest plot.	
[redacted]		[redacted]	

ts	21	Present results of each meta-analysis done, including confidence intervals and measures of consistency.	

[redacted]

s studies	22	Present results of any assessment of risk of bias across studies (see Item 15).	

[redacted]

s	23	Give results of additional analyses, if done (e.g., sensitivity or subgroup analyses, meta-regression [see Item 16]).	
[redacted]			

[redacted]

ence	24	Summarize the main findings including the strength of evidence for each main outcome; consider their relevance to key groups (e.g., healthcare providers, users, and policy makers).
[redacted]		[redacted]

	25	Discuss limitations at study and outcome level (e.g., risk of bias), and at review-level (e.g., incomplete retrieval of identified research, reporting bias).	
[redacted]		[redacted]	

	26	Provide a general interpretation of the results in the context of other evidence, and implications for future research.	

[redacted]

[redacted]

	27	Describe sources of funding for the systematic review and other support (e.g., supply of data); role of funders for the systematic review.	
[redacted]			

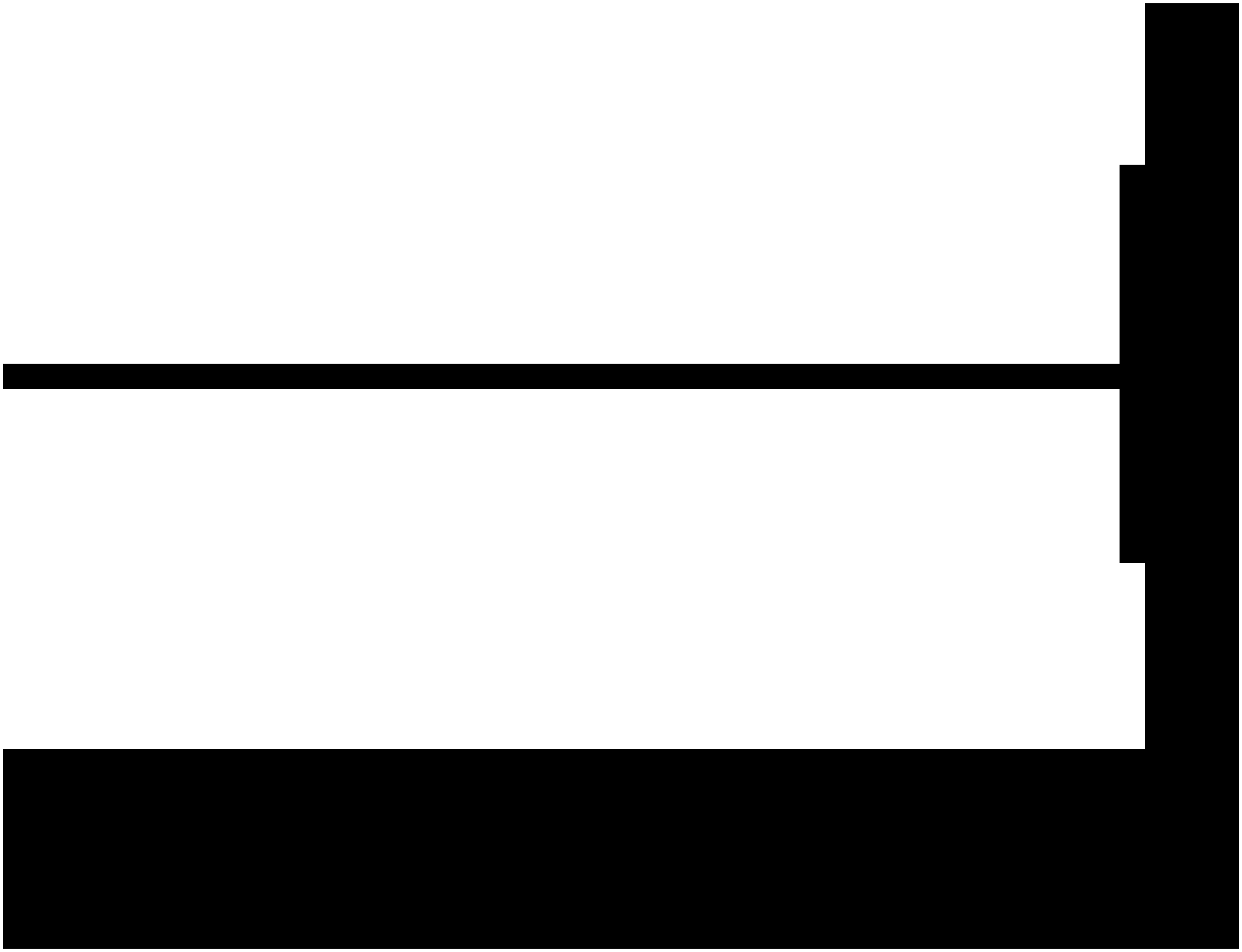

From: Moher D, Liberati A, Tetzlaff J, Altman DG, The PRISMA Group (2009). Preferred Reporting Items for Systematic Reviews and Meta-Analyses: The PRISMA Statement. PLoS Med 6(6): e1000097. doi:10.1371/journal.pmed1000097

For more information, visit: **www.prisma-statement.org**.

[redacted]

Supplemental Table 2. Search strategy

Databases	Search terms	Numbers of records
Pubmed	(((((((DRB1) OR MHC) OR major histocom-patibility complex) OR HLA) OR human leukocyte antigen) OR "HLA Antigens"[Mesh])) AND (((((((nonscarring hair loss) OR ophiasis) OR alopecia celsi) OR alopecia universalis) OR alopecia totalis) OR Alopecia Areata) OR "Alopecia Areata"[Mesh])	177
Cochrane Library	#1 MeSH descriptor: [Alopecia Areata] explode all trees<br>#2 nonscarring hair loss<br>#3 ophiasis<br>#4 alopecia celsi<br>#5 alopecia universalis<br>#6 alopecia totalis	2

	#7 Alopecia Areata<br>#8 #1 or #2 or #3 or #4 or #5 or #6 or #7<br>#9 MeSH descriptor: [HLA Antigens] explode all trees<br>#10 human leukocyte antigen<br>#11 HLA<br>#12 major histocompatibility complex<br>#13 MHC<br>#14 DRB1<br>#15 #9 or #10 or #11 or #12 or #13 or #14<br>#16 #8 and #15	
Embase	#1 Alopecia Areata/ or alopecia areata.mp.<br>#2 nonscarring hair loss.mp.<br>#3 ophiasis.mp.<br>#4 alopecia celsi.mp.<br>#5 alopecia universalis.mp.<br>#6 alopecia totalis.mp.<br>#7 #1 or #2 or #3 or #4 or #5 or #6<br>#8 HLA Antigens.mp.<br>#9 human leukocyte antigen<br>#10 HLA<br>#11 major histocompatibility complex<br>#12 MHC<br>#13 DRB1<br>#14 #8 or #9 or #10 or #11 or #12 or #13<br>#15 #7 and #14	285
CBM	(((((((("斑秃"[常用字段:智能]) OR "油风"[常用字段:智能]) OR "鬼剃头"[常用字段:智能]) OR "鬼舐头"[常用字段:智能]) OR "秃病"[常用字段:智能]) OR "秃发"[常用字段:智能]) OR "脱发"[常用字段:智能]) OR "脱发症"[常用字段:智能]) AND "人白细胞抗原"[常用字段:智能])	45
WANGFANG	('油风' or '斑秃' or '鬼舐头' or '鬼剃头' or '秃病' or '秃发' or '脱发' or '脱发症') and ('HLA' or '人白细胞抗原' or '人类白细胞抗原')	56
CNKI	SU=('油风'+'斑秃'+'鬼舐头'+'鬼剃头'+'秃病'+'秃发'+'脱发'+'脱发症') and SU=('HLA'+'人白细胞抗原'+'人类白细胞抗原')	42
VIP	题名或关键词=油风+斑秃+鬼舐头+鬼剃头+秃病+秃发+脱发+脱发症 与 题名或关键词=HLA+hla+人白细胞抗原+人类白细胞抗原	19

Supplemental Table 3. Excluded Studies.

Study(Author,year)	Citation	Reason(s) for Exclusion
Valsecchi 1985	Acta Derm Venereol, 1985, 65(2), 175-177	Familial study with insufficient genetic data
Orecchia 1987	Dermatologica, 1987, 175(1), 10-14	HLA typing was insufficient
Morling 1991	Dis Markers, 1991, 9(1), 35-42	Only HLA-DQ alleles were characterized
Colombe 1995	J Am Acad Dermatol, 1995, 33(5 Pt 1), 757-764	HLA typing was insufficient and study design was not case-control
Colombe 1995	J Invest Dermatol, 1995, 104(5 Suppl), 4S-5S	HLA typing was insufficient and study design was not case-control
Price 1996	ermatol Clin, 1996, 14(4), 679-689	HLA typing was insufficient and study design was not case-control
Colombe 1999	J Investig Dermatol Symp Proc, 1999, 4(3), 216-219	HLA typing was insufficient and study design was not case-control
Xiao 2006	J Dermatol Sci, 2006, 41(2), 109-119	Only HLA-A,-B, and-C alleles were typed
Petukhova 2010	Nature, 2010, 466(7302), 113-117	HLA typing was insufficient
Zhang 2010	International Journal of Blood Transfusion and Hematology	HLA typing was insufficient
Haida 2013	Immunogenetics, 2013, 65(7), 553-557	Only HLA -C alleles were typed

Supplemental Table 4. Scores of Newcastle-Ottawa Scale

NO	First Authors	Selection				Comparability	Exposure			Total
		①	②	③	④	⑤	⑥	⑦	⑧	
1	Akar	1	0	0	1	0	1	1	0	4
2	Barahmani	1	0	0	1	0	1	1	0	4
3	Aliagaoglu	1	0	0	1	0	1	1	0	4
4	Megiorni	1	0	1	1	0	1	1	0	5
**1.1.1 5**	**1.1.2 Broniarczyk-Dyla**	1	0	0	1	0	1	1	0	4
6	Marques	1	0	0	1	0	1	1	0	4
7	Qi	1	1	0	1	2	1	1	0	7
8	Tao	1	1	0	1	0	1	1	0	5

**1.1.3 9**	**1.1.4 Entz**	1	0	0	1	2	1	1	0	6
**1.1.5 10**	Zhang	1	1	0	1	0	1	1	0	5
**1.1.6 11**	Attia	1	1	0	1	2	1	1	0	7
**1.1.7 12**	Barbosa	1	0	0	1	2	1	1	0	6

①Is the case definition adequate? ②Representativeness of the cases ③Selection of Controls ④Definition of Controls ⑤Comparability of cases and controls on the basis of the design or analysis⑥Ascertainment of exposure ⑦Same method of ascertainment for cases and controls⑧Non-Response rate

Cochran WG. The combination of estimates from different experiments. Biometrics. 1954; 10:101-129.
